# A Randomized Controlled Trial Comparing the Effects of Counseling and Alarm Device on HAART Adherence and Virologic Outcomes

**DOI:** 10.1371/journal.pmed.1000422

**Published:** 2011-03-01

**Authors:** Michael H. Chung, Barbra A. Richardson, Kenneth Tapia, Sarah Benki-Nugent, James N. Kiarie, Jane M. Simoni, Julie Overbaugh, Mena Attwa, Grace C. John-Stewart

**Affiliations:** 1 Department of Global Health, University of Washington, Seattle, Washington, United States of America; 2 Department of Medicine, University of Washington, Seattle, Washington, United States of America; 3 Department of Epidemiology, University of Washington, Seattle, Washington, United States of America; 4 Department of Biostatistics, University of Washington, Seattle, Washington, United States of America; 5 Division of Public Health Sciences, Fred Hutchinson Cancer Research Center, Seattle, Washington, United States of America; 6 Department of Obstetrics and Gynaecology, University of Nairobi, Nairobi, Kenya; 7 Department of Psychology, University of Washington, Seattle, Washington, United States of America; 8 Division of Human Biology, Fred Hutchinson Cancer Research Center, Seattle, Washington, United States of America; 9 Coptic Hospital, Nairobi, Kenya; McMaster University, Canada

## Abstract

**Background:**

Behavioral interventions that promote adherence to antiretroviral medications may decrease HIV treatment failure. Antiretroviral treatment programs in sub-Saharan Africa confront increasing financial constraints to provide comprehensive HIV care, which include adherence interventions. This study compared the impact of counseling and use of an alarm device on adherence and biological outcomes in a resource-limited setting.

**Methods and Findings:**

A randomized controlled, factorial designed trial was conducted in Nairobi, Kenya. Antiretroviral-naïve individuals initiating free highly active antiretroviral therapy (HAART) in the form of fixed-dose combination pills (d4T, 3TC, and nevirapine) were randomized to one of four arms: counseling (three counseling sessions around HAART initiation), alarm (pocket electronic pill reminder carried for 6 months), counseling plus alarm, and neither counseling nor alarm. Participants were followed for 18 months after HAART initiation. Primary study endpoints included plasma HIV-1 RNA and CD4 count every 6 months, mortality, and adherence measured by monthly pill count. Between May 2006 and September 2008, 400 individuals were enrolled, 362 initiated HAART, and 310 completed follow-up. Participants who received counseling were 29% less likely to have monthly adherence <80% (hazard ratio [HR] = 0.71; 95% confidence interval [CI] 0.49–1.01; *p = *0.055) and 59% less likely to experience viral failure (HIV-1 RNA ≥5,000 copies/ml) (HR 0.41; 95% CI 0.21–0.81; *p = *0.01) compared to those who received no counseling. There was no significant impact of using an alarm on poor adherence (HR 0.93; 95% CI 0.65–1.32; *p = *0.7) or viral failure (HR 0.99; 95% CI 0.53–1.84; *p = *1.0) compared to those who did not use an alarm. Neither counseling nor alarm was significantly associated with mortality or rate of immune reconstitution.

**Conclusions:**

Intensive early adherence counseling at HAART initiation resulted in sustained, significant impact on adherence and virologic treatment failure during 18-month follow-up, while use of an alarm device had no effect. As antiretroviral treatment clinics expand to meet an increasing demand for HIV care in sub-Saharan Africa, adherence counseling should be implemented to decrease the development of treatment failure and spread of resistant HIV.

**Trial registration:**

ClinicalTrials gov NCT00273780

*Please see later in the article for the Editors' Summary*

## Abstract 2

Michael Chung and colleagues show that intensive early adherence counseling at HAART initiation resulted in sustained, significant impact on adherence and virologic treatment failure, whereas use of an alarm device had no effect.

## Editors' Summary

**Background:**

Adherence to HIV treatment programs in poor countries has long been cited as an important public health concern, especially as poor adherence can lead to drug resistance and inadequate treatment of HIV. However, two factors have recently cast doubt on the poor adherence problem: (1) recent studies have shown that adherence is high in African HIV treatment programs and often better than in Western HIV clinics. For example, in a meta-analysis of 27 cohorts from 12 African countries, adequate adherence was noted in 77% of subjects compared to only 55% among 31 North America cohorts; (2) choice of antiretroviral regimens may impact on the development of antiretroviral resistance. In poor countries, most antiretroviral regimens contain non-nucleoside reverse transcriptase inhibitors (NNRTIs), such as nevirapine or efavirenz, which remain in the patient's circulation for weeks after single-dose administration. This situation means that such patients may not experience antiretroviral resistance unless they drop below 80% adherence—contrary to the more stringent 95% plus adherence levels needed to prevent resistance in regimens based on unboosted protease inhibitors—ultimately, off-setting some treatment lapses in resource-limited settings where NNRTI-based regimens are widely used.

**Why Was This Study Done?:**

Given that adherence may not be as crucial an issue as previously thought, antiretroviral treatment programs in sub-Saharan Africa may be spending scarce resources to promote adherence to the detriment of some potentially more effective elements of HIV treatment and management programs. Although many treatment programs currently include adherence interventions, there is limited quality evidence that any of these methods improve long-term adherence to HIV treatment. Therefore, it is necessary to identify adherence interventions that are inexpensive and proven to be effective in resource-limited settings. As adherence counseling is already widely implemented in African HIV treatment programs and inexpensive alarm devices are thought to also improve compliance, the researchers compared the impact of adherence counseling and the use of an alarm device on adherence and biological outcomes in patients enrolled in HIV programs in rural Kenya.

**What Did the Researchers Do and Find?:**

The researchers enrolled 400 eligible patients (newly diagnosed with HIV, never before taken antiretroviral therapy, aged over 18 years) to four arms: (1) adherence counseling alone; (2) alarm device alone; (3) both adherence counseling and alarm device together; and (4) a control group that received neither adherence counseling nor alarm device. The patients had blood taken to record baseline CD4 count and HIV-1 RNA and after starting HIV treatment, returned to the study clinic every month with their pill bottles for the study pharmacist to count and recorded the number of pills remaining in the bottle, and to receive another prescription. Patients were followed up for 18 months and had their CD4 count and HIV-1 RNA measured at 6, 12, and 18 months.

Patients receiving adherence counseling were 29% less likely to experience poor adherence compared to those who received no counseling. Furthermore, those receiving intensive early adherence counseling were 59% less likely to experience viral failure. However, there was no significant difference in mortality or significant differences in CD4 counts at 18 months follow-up between those who received counseling and those who did not. There were no significant differences in adherence, time to viral failure, mortality, or CD4 counts in patients who received alarm devices compared to those who did not.

**What Do These Findings Mean?:**

The results of this study suggest that intensive adherence counseling around the time of HIV treatment initiation significantly reduces poor adherence and virologic treatment failure, while using an alarm device has no effect. Therefore, investment in careful counseling based on individual needs at the onset of HIV treatment initiation, appears to have sustained benefit, possibly through strengthening the relationship between the health care provider and patient through communication, education, and trust. Interactive adherence counseling supports the bond between the clinic and the patient and may result in fewer patients needing to switch to expensive second-line medications and, possibly, may help to decrease the spread of resistant HIV. These findings define an adherence counseling protocol that is effective and are highly relevant to other HIV clinics caring for large numbers of patients in sub-Saharan Africa.

**Additional Information:**

Please access these Web sites via the online version of this summary at http://dx.doi.org/10.1371/journal.pmed.1000422.

- UNAIDS provides information about HIV treatment strategies
- The American Public Health Association has information about adherence to HIV treatment regimens
- The US Department of Health and Human Services has information for patients about adherence to HIV treatment
- The World Health Organization provides information about HIV treatment pharmacovigilance

## Introduction

The introduction of antiretroviral medications on a public health scale to treat HIV-positive persons in sub-Saharan Africa has been accompanied by concern that these actions will result in widespread viral resistance because of poor adherence [1]. However, recent studies have shown that adherence is high in African HIV treatment programs and often better than in Western HIV clinics [2],[3]. In a meta-analysis of 27 cohorts from 12 African countries, adequate adherence was noted in 77% of participants compared to only 55% among 31 North America cohorts [4].

Among individuals with imperfect adherence, the choice of antiretroviral regimens may impact the development of antiretroviral resistance. In resource-limited settings, most antiretroviral regimens contain non-nucleoside reverse transcriptase inhibitors (NNRTI) [5]–[7]. NNRTI drugs such as nevirapine or efavirenz have a long half-life in vivo; the concentrations of nevirapine in plasma may remain for weeks after single-dose administration [8]. Thus, antiretroviral resistance may not occur in patients on NNRTI regimens until their adherence drops to intermediate levels below 80% [9]. This adherence differs significantly from the more stringent adherence requirements (≥95%) of regimens based on unboosted protease inhibitors (PI) [10]–[13], and may allow more lapses to occur in resource-limited settings where NNRTI-based regimens are widely used.

Given these findings, antiretroviral treatment programs in sub-Saharan Africa may be spending valuable resources promoting adherence [14],[15]. Targeted adherence interventions require trained staff, space to accommodate confidentiality, and time on the part of the patient and counselor. In a rapidly growing, large-scale treatment program, these efforts could be a costly, rate-limiting step in the enrollment of newly diagnosed HIV-positive patients requiring care [16],[17]. Although many antiretroviral treatment programs in this setting include adherence interventions with medications [18]–[20], there is limited quality evidence that any of these methods improve long-term adherence to highly active antiretroviral therapy (HAART) [21]–[24]. This information is essential as HIV clinics in sub-Saharan Africa are forced to deliver antiretroviral treatment with increasingly limited funding [25]–[27].

Inexpensive adherence interventions need to be identified that are proven to be effective in resource-limited settings. Counseling is widely implemented in HIV treatment programs in Africa, but delivery of this intervention is not uniform and its impact on improving adherence is unclear. There is some evidence that early intensive counseling around the time of HAART initiation may be beneficial [28]. In addition, it has been suggested that alarm devices, which are relatively inexpensive and easy to distribute, may improve pill adherence in Kenya [29]. In order to concurrently evaluate the value of these distinct adherence interventions in a resource-limited setting, we conducted a factorial randomized trial in Nairobi, Kenya and compared impact of adherence counseling and the use of an alarm device on adherence and biological outcomes.

## Methods

### Setting and Recruitment

The trial was conducted between May 2006 and September 2008 at the Coptic Hope Center for Infectious Diseases in Nairobi, Kenya (Texts S1 and S2). The Hope Center is an HIV treatment clinic established in 2004 by the University of Washington and the Coptic Orthodox Mission. Supported by the President's Emergency Plan for AIDS Relief (PEPFAR), the Hope Center provides free HIV care and antiretroviral treatment. Clinic procedures have been described elsewhere [30]. As per Kenyan national guidelines, newly diagnosed clients are initiated on free NNRTI-based HAART if they have: a CD4 count <250 cells/mm^3^, World Health Organization (WHO) clinical stage IV disease, or a CD4 count <350 cells/mm^3^ with WHO clinical stage III disease [31]. Individuals were eligible to enroll in the study if they were ≥18 y of age, antiretroviral naïve, agreed to home visits, and planned to live in Kenya for at least 2 y.

### Design

The study evaluated two interventions based on models of cognitive and behavioral theory to promote adherence to antiretroviral medications: intensive adherence counseling and use of a pocket alarm device [32],[33]. In a 2×2 factorial design, participants were randomized in a 1∶1∶1∶1 ratio to one of four arms prior to initiating HAART: (1) adherence counseling alone; (2) alarm device alone; (3) both adherence counseling and alarm device together; and (4) a control group that received neither adherence counseling nor alarm device. Randomization was performed at enrollment by the study nurse who opened a sealed envelope containing a computer-generated block randomization code that was developed by the study biostatistician. Study investigators and participants were not blinded to the interventions. The prespecified primary endpoints for this study were adherence as measured by monthly manual pill counts, plasma HIV-1 RNA, and CD4 count.

### Interventions

In the adherence counseling intervention, trained counselors administered two counseling sessions to participants prior to HAART initiation and a third session one month after HAART initiation (Text S1). Counseling sessions around HAART initiation were based on a model of successful antiretroviral adherence promotion at a large University of Washington-affiliated HIV treatment program in Seattle, Washington [28]. All counseling sessions followed a written standardized protocol and lasted between 30 and 45 min. In the first session, counselors explored personal barriers to good adherence and taught participants about the HIV, the virus that causes AIDS, antiretroviral medications, and the risks of treatment failure due to poor adherence. The second session occurred on a separate day and involved a review of a participant's understanding and readiness to begin antiretroviral medications. The third session allowed the counselor to examine practical and personal issues that the participant may have encountered on HAART. The adherence counseling intervention had been previously used and adapted at the same site in Kenya for over 2 y and was delivered in English and Kiswahili.

Participants in the alarm device intervention received a small pocket digital alarm, the ALRT PC200 (ALR Technologies Inc), which the individual was to carry at all times for 6 mo duration. The device was programmed by the study staff to beep and flash twice a day at a time convenient to the participant when medications were to be taken. The digital alarm could not be reprogrammed or inactivated by the individual and was utilized for 6 mo after HAART initiation before being disabled by study staff.

### Control

At HAART initiation, the study pharmacist explained the side effects of medications and problems associated with poor adherence in a 15-min session prior to dispensing drugs. All participants, including those in the control arm, received this educational message. Participants randomized to the control group did not receive adherence counseling or an alarm device.

### Follow-up

At enrollment, participants signed a written informed consent, had blood drawn for HIV-1 RNA levels and CD4 count, and shared information on sociodemographic characteristics, risk behavior, and distance from clinic. After initiating HAART, participants returned to the study clinic at monthly intervals with their pill bottles to pick up antiretroviral medications. At each monthly visit, the study pharmacist counted and recorded the number of pills remaining in the bottle, the visit date, whether the participant took his or her morning dose, and the number of pills dispensed that day. Participants randomized to receive an alarm device were asked if they had been using the device at each of these monthly visits, and any defective, lost, or stolen devices were replaced at this time. Blood was drawn for CD4 count and HIV-1 RNA at 6, 12, and 18 mo after HAART initiation. Participants were followed for 18 mo on HAART before exiting the study. HIV-1 RNA was measured using the Gen-Probe quantitative HIV-1 viral load assay [34]. CD4 counts were determined using flow cytometry (FACScan, Becton Dickinson).

### Endpoints

Adherence was calculated at each pharmacy refill visit as the percentage of dispensed doses that were taken since the previous visit to the study pharmacy. Total time between the last date the participant was in the pharmacy and the participant's subsequent pharmacy visit included any missed visits to the pharmacy or time when the participant was not attending the clinic. This calculated adherence was assumed to be constant and the same as daily adherence throughout this time period. Continuous adherence and time to monthly adherence <80% and <95% were calculated [10]–[13]. Viral failure was defined as the first plasma HIV-1 RNA level ≥5,000 copies/ml measured at least 4 mo after HAART initiation [35]. Other study endpoints included mortality and change in CD4 count.

### Statistical Methods

All analyses were modified intent-to-treat in that only individuals who initiated HAART, and therefore had pill count data collected for adherence measurement, were included in the analyses. Data from all participants were utilized in analyses until time of exit either because of completion of the study, loss to follow-up, or death. Because of the factorial design of the study, models to evaluate the study interventions were developed by first testing the statistical interaction between those who received counseling and those who received alarm. In all models, the interaction term had a *p*-value larger than 0.20 and therefore was dropped from the model. Accordingly, following the factorial design of the study, those who received counseling were compared against those who received no counseling and those who received an alarm device were compared against those who received no alarm. Analyses of discrete time-to-viral-failure event models were performed using SAS 9.2; all other analyses were performed using StataSE v10 (StataCorp).

Univariate comparisons were done using the Mann Whitney U test for continuous variables, and Chi square tests for binary variables. Baseline characteristics were compared between interventions and if there were statistically significant differences (i.e., an imbalance in a variable at baseline), then these variables were tested to see if they also related to the various outcomes of interest (adherence, mortality, viral load, and CD4 count). If baseline variables met both criteria (imbalanced at baseline and related to outcome), then they were included in a multivariable model [36]. When comparing counseling versus no counseling, the only variable that met both criteria was age in the analysis of CD4 count. When comparing alarm device versus no alarm device, the only variables that met these criteria were ever having given or received money/favors in exchange for sex in the adherence analyses and baseline plasma viral load in the viral load and mortality analyses.

Analyses of time to monthly adherence <80% and <95%, viral failure, and death were performed using Cox proportional hazards models. Kaplan-Meier methods were used to create time to event graphs. Longitudinal analyses of adherence were performed using generalized estimating equations (GEE) with an exchangeable correlation matrix and robust variance estimate. Linear regression was used to assess differences in change in CD4 count.

The sample size was calculated as follows. Given information from previous studies [3],[29],[37], it was estimated that approximately 60% of participants would maintain adherence ≥95% without intervention and that the interventions would help 82%–94% of the participants maintain ≥95% adherence. Assuming a two-sided test with α = 0.05, 74 participants were needed in each arm to have 80% power to detect a 22% difference (60% versus 82%). One hundred participants were randomized to each arm to allow for loss to follow-up and mortality.

### Ethical Review

The study protocol was reviewed and approved by the institutional review boards at the University of Washington and Kenyatta National Hospital (Nairobi, Kenya).

## Results

### Study Population

**Figure 1:**
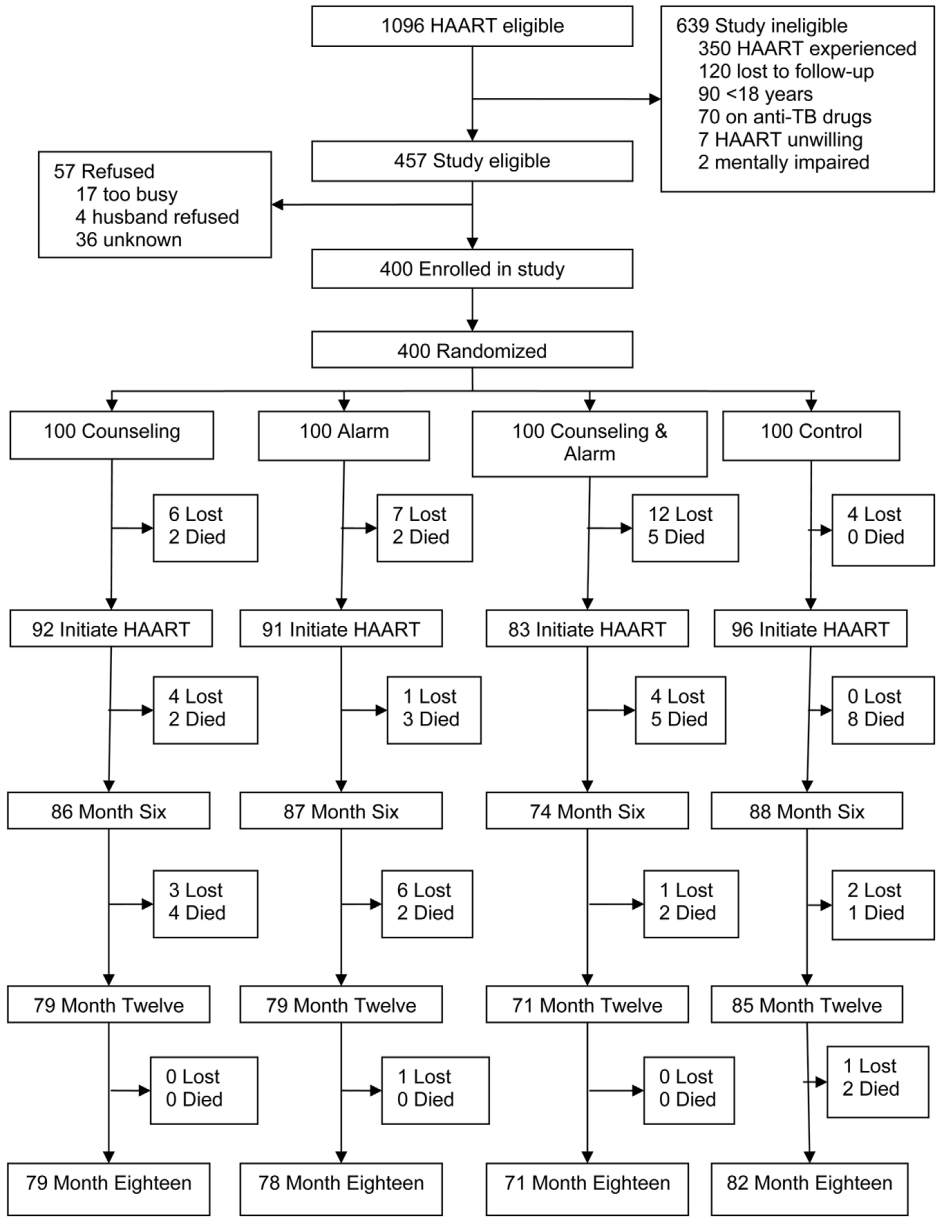
Trial profile. TB, tuberculosis.

Enrollment of participants began on May 2006 and ended on November 2006 during which time 1,096 patients were deemed eligible to receive HAART at the Hope Center (Figure 1). Of these patients, 457 were eligible to participate in the study and 400 accepted study enrollment. The 400 participants were randomized to one of four study arms with each arm containing 100 participants. 639 patients were ineligible for the study because they were HAART experienced (55%), lost to follow-up before study recruitment (19%), under 18 y of age (14%), on antituberculosis medications (11%), unwilling to initiate HAART (1%), or mentally impaired (<1%). Study follow-up was completed in September 2008 as per the study protocol.

362 participants initiated HAART (a generic fixed-dose combination pill containing d4T, 3TC, and nevirapine) and were included in analyses of the study endpoints (Table 1). In this study population, 66% were female, the median age was 36 y (interquartile range [IQR] 31–42), the median monthly rent was US$28 (IQR 11–56), the median distance from home to clinic was 10 kilometers (IQR 6–15), and 10% had ever given or received money/favors in exchange for sex. Of the 362 HAART initiators: 347 (96%) visited the study pharmacy at least two times and consequently could have adherence calculated; 359 (99%) had plasma viral levels collected at enrollment; and 331 (91%) had at least one follow-up CD4 count measurement. The mean adherence for the 347 participants who had adherence calculated was 92.5% (95% confidence interval (CI) 91.2–94.0) and the median adherence was 97.3% (IQR 93.5–98.7); 314 participants (90.5%) had overall adherence ≥80%; and 237 (68.3%) had overall adherence ≥95%.

**Table 1:** Baseline characteristics among 362 HAART initiators.

Characteristic	Counseling (*n = *92)	Alarm (*n = *91)	Counseling and Alarm (*n = *83)	Control (*n = *96)
Age, y	36 (31–44)	36 (32–41)	38 (32–44)	35 (30–40)
*n* (%) female	54 (59)	62 (68)	55 (66)	68 (71)
Education, y	12 (8–14)	12 (8–14)	11 (8–13)	12 (8–13)
*n* (%) unemployed	31 (34)	27 (30)	22 (27)	40 (42)
*n* (%) married or attached	43 (47)	50 (55)	42 (51)	51 (53)
Monthly rent, US$	28 (13–56)	25 (11–70)	23 (10–56)	28 (11–56)
*n* (%) flush toilet	47 (51)	41 (45)	35 (42)	39 (41)
Individuals living in household	4 (3–5)	3 (2–5)	4 (2–5)	4 (3–5)
*n* (%) cost of travel to clinic ≥US$.70*	59 (64)	49 (54)	62 (75)	52 (54)
Distance from home to clinic, kilometer**	11 (7–15)	9 (5–15)	11 (8–16)	10 (6–13)
Age at first sex, y	18 (16–20)	18 (16–20)	18 (16–20)	18 (16–20)
Lifetime sexual partners	4 (2–8)	4 (2–5)	4 (3–8)	4 (2–6)
*n* (%) ever exchanged money or favors for sex	12 (13)	5 (6)	7 (8)	13 (14)
Plasma HIV-1 viral load, copies/ml	627,200 (202,300–1,349,200)	402,050 (161,200–782,600)	441,600 (95,100–1,047,200)	473,200 (234,700–1,264,650)
CD4 count, cells/ml	113 (63–171)	115 (46–190)	131 (70–190)	114 (67–173)

Data are median (range), unless otherwise indicated.

*p = 0.01

**p = 0.05

For all endpoint analyses, the interaction term between counseling and alarm was tested and not statistically significant. Therefore, those who received counseling (participants from the counseling and counseling plus alarm arms) were compared to those who received no counseling (participants from the alarm and control arms) and those who received an alarm device (participants from the alarm and counseling plus alarm arms) were compared to those who received no alarm (participants from the counseling and control arms).

### Loss to Follow-up

At the end of study follow-up, there were 52 patients who were lost to follow-up and 38 deaths (Figure 1). In a comparison of baseline characteristics, such as age, gender, income, education, distance, and sexual behavior, there were no significant differences between those lost and those retained except the former were more likely to have a higher rent (median US$, 46 versus 25; *p = *0.002) and fewer people per household (3 versus 4; *p = *0.02). Prior to HAART initiation, 29 (7%) participants were lost to follow-up and nine (2%) died. Monthly rent was higher among those who did not initiate HAART compared to those who did (median US$, 42 versus 28; *p = *0.03).

### Intervention Participation

200 participants were randomized to the adherence counseling intervention and 164 (82%) received all three assigned counseling sessions, 21 (10.5%) received two sessions, seven (3.5%) received one session, and eight (4%) did not undergo any sessions. All of those who did not receive all three counseling sessions died or were lost to follow-up before completing the intervention.

200 participants were randomized to receive the alarm device intervention and to use it for 6 mo after HAART initiation. 29 individuals died or were lost to follow-up before the pocket alarm was used. Among those who used the external reminder, 150 (88%) reported using it for 5 to 6 mo after HAART initiation: 107 (63%) reported using the alarm at all monthly follow-up visits over 6 mo, 43 (25%) reported not using the device at one visit, 11 (6%) at two visits, seven (4%) at three visits, and three (2%) at four visits. Reasons for not using the pocket alarm included: the device not working properly (72%), stolen (11%), lost (6%), no reason (6%), forgot (2%), no need (2%), and unable (1%). There were no reports of not using an alarm device because of stigma and all participants requested to keep the inactivated alarm device for use as a pocket watch after the 6-month intervention had ended.

There was no harm or unintended effect recorded for either intervention.

### Counseling

**Figure 2:**
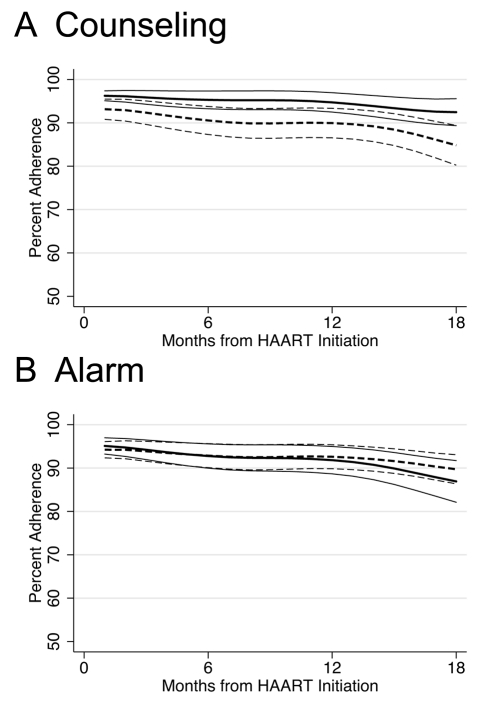
Lowess curves of percent adherence over time in months since HAART initiation by intervention. Thick lines indicate average percent adherence. Thin lines indicate 95% CIs. Solid lines indicate intervention. Dashed lines indicate no intervention. (A) Counseling versus no counseling. (B) Alarm versus no alarm.

**Figure 3:**
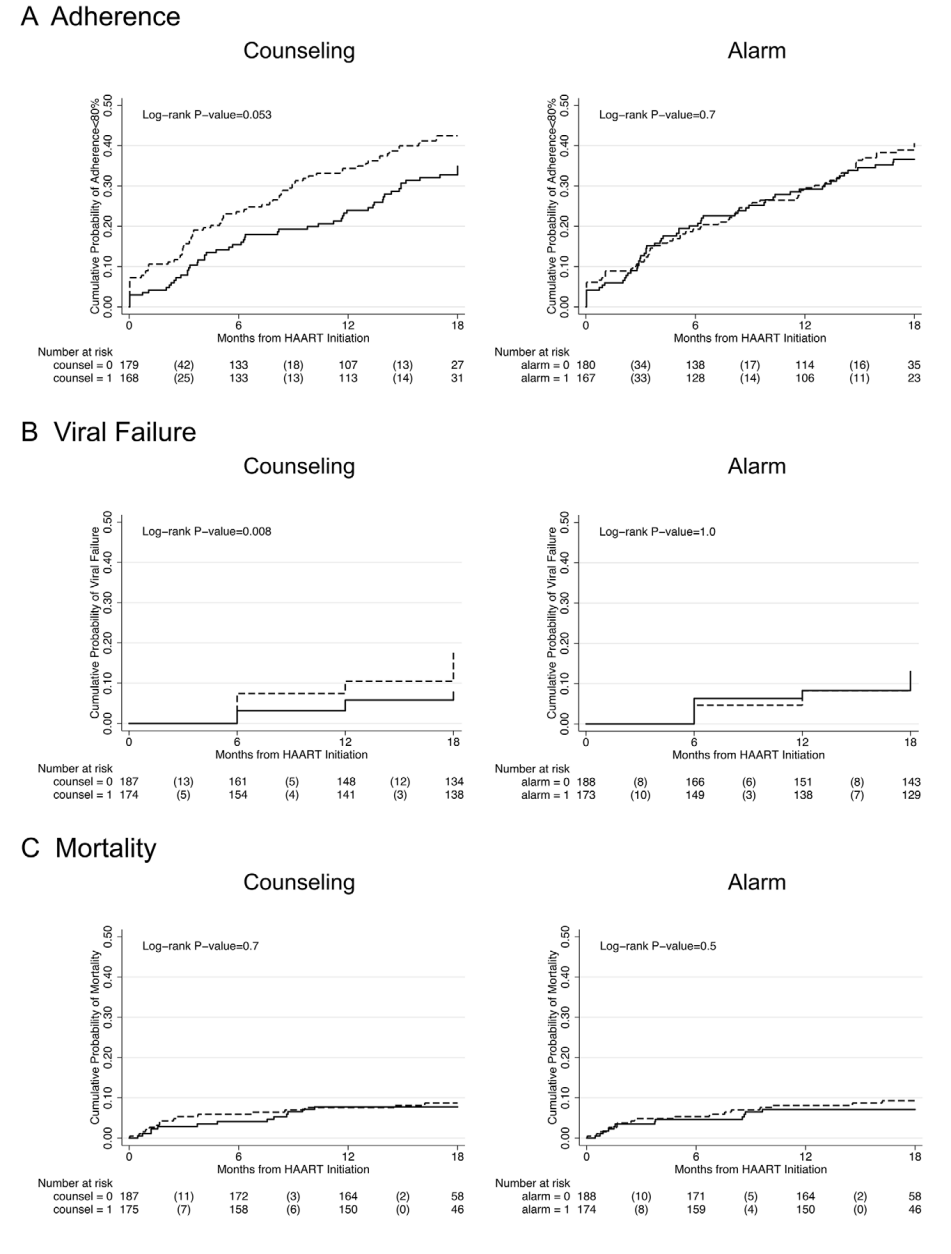
Kaplan-Meier survival curves comparing counseling versus no counseling and alarm versus no alarm. Study outcomes include (A) adherence <80%, (B) viral failure (≥5,000 copies/ml), and (C) mortality. Solid line indicates intervention and dashed line indicates no intervention. The number of participants at risk at each time point is listed below the graph with the number of failure events in parentheses.

In longitudinal analysis, adherence during the first month after initiating HAART was significantly higher among those who received counseling (difference in intercepts, 3.58%; 95% CI 0.50%–6.66%; *p = *0.023) compared to those who did not receive counseling, and this difference was constant over 18 mo follow-up (difference in slopes, 0.13% per month; 95% CI −0.16% to 0.42%; *p = *0.4) (Figure 2A). Those participants receiving counseling were on the margin of significance for being 29% less likely to experience monthly adherence <80% over 18 mo follow-up compared to those who received no counseling (hazard ratio [HR] 0.71; 95% CI 0.49–1.01; *p = *0.055) (Figure 3A; K-M log-rank *p = *0.053). No significant differences were found in adherence <95% between those who received counseling and those who did not (HR 0.89; 95% CI 0.70–1.12; *p = *0.3).

Participants who received counseling were 59% less likely to experience viral failure (HIV-1 RNA ≥5,000 copies/ml) than those who did not receive counseling (HR 0.41; 95% CI 0.21–0.81; *p = *0.01) (Figure 3B). Significant differences were also found after setting viral failure at a lower threshold of HIV-1 RNA ≥1,000 copies/ml (HR 0.45; 95% CI 0.24–0.82; *p = *0.01).

There was no significant difference in mortality between those who received counseling and those who did not (HR 0.87; 95% CI 0.42–1.81; *p = *0.7) (Figure 3C). There was also no significant difference in CD4 count increase at 18 mo follow-up between those who received counseling and those who did not (median CD4 cells/ml increase, 202 versus 225; *p = *0.7), and this lack of significant difference remained in multivariable modeling controlling for age at baseline (unpublished data).

### Alarm Device

There was no significant difference in any of the study endpoints between those who received alarm device and those who did not (Figure 3). In longitudinal analysis, adherence during the first month after initiating HAART did not differ between those who received an alarm device and those who did not (difference in intercepts, 0.77%; 95% CI −2.36% to 3.89%; *p = *0.6), and this lack of difference continued over 18 mo follow-up (difference in slopes,−0.15% per month; 95% CI −0.45% to 0.15%; *p = *0.3) (Figure 2B). Those who received an alarm device were no less likely to experience monthly adherence <80% over 18 mo follow-up compared to those who received no alarm device (HR 0.93; 95% CI 0.65–1.32; *p = *0.7), even after adjusting for baseline differences of ever having given or received money/favors in exchange for sex (adjusted hazard ratio [aHR] 0.95; 95% CI 0.66–1.35; *p = *0.8]. In addition, there was no significant difference in time to viral failure ≥5,000 copies/ml (HR 0.99; 95% CI 0.53–1.84; *p = *1.0), death (HR 0.76; 95% CI 0.36–1.60; *p = *0.5), immunological recovery (median CD4 count cells/ml increase, 197 versus 227; *p = *0.2), and adherence <95% (HR 0.97; 95% CI 0.77–1.22; *p = *0.8) between those who received an alarm device and those who did not. These results remained unchanged in multivariable analyses and at a viral failure threshold of HIV-1 RNA ≥1,000 copies/ml (unpublished data).

## Discussion

In this randomized controlled trial comparing counseling and the use of an alarm device to improve adherence to antiretroviral medications in Kenya, participants receiving intensive early adherence counseling were 59% (HR 0.41; 95% CI 0.21–0.81; *p = *0.01) less likely to experience viral failure, demonstrating the powerful impact of this behavioral intervention on biological outcomes. Although poor adherence has been associated with plasma HIV-1 viral rebound and development of viral resistance [38],[39], few trials have demonstrated an association between an intervention designed to improve adherence and virologic impact [37],[40]–[42]. This study found a significant association between having received adherence counseling and antiretroviral treatment failure as defined by the WHO [35].

Those receiving adherence counseling in this study were 29% (HR 0.71; 95% CI 0.49–1.01; *p = *0.055) less likely to experience poor adherence compared to those who received no counseling. The positive effects of counseling on adherence in this study were found immediately after HAART initiation and were sustained over 18 mo. Trained counselors provided not only didactic information but discussed barriers to good adherence and developed a relationship with participants through one-on-one interviews conducted over 2 mo. Motivating individuals to change their behavior through counseling appears to give individuals tools they need to improve their adherence [43]. Dedicating time towards effective communication regarding adherence may strengthen a provider-patient relationship that, in turn, promotes adherence through trust [44]. These findings support the implementation of adherence counseling among HIV clinics in sub-Saharan Africa and suggest that forms of relationship-strengthening adherence interventions should be continued, even in the setting of increasing resource constraints.

An intervention that reduces viral failure by more than half represents significant cost savings both from the deferred purchase of more expensive second-line antiretroviral medications and the potential expense of treating opportunistic reinfections. Compared to the costs of treatment failure, employing counselors in this setting is relatively inexpensive. Using tents where necessary to preserve confidentiality and having one counselor interact with groups of patients in the first session further decreases costs. Thus, upfront investment in adherence counseling could save programs from future financial losses due to treatment failure that, without counseling, could double. Our study also demonstrates that the best method of “treatment preparedness” as recommended by the WHO [35], is not simply educating patients about antiretroviral drugs, side effects, and adherence, as was given to all participants in this study. Instead, two interactive, anticipatory sessions of counseling prior to HAART and a session 1 mo after HAART initiation may better prepare a patient in a resource-limited setting to adhere to a lifetime of antiretroviral drugs.

In contrast to counseling, our study did not find any beneficial effect of using an external alarm device on adherence or viral failure. This result concurs with recent studies and reviews that find limited impact or insufficient evidence to demonstrate the effectiveness of using an external reminder alone [40],[45],[46]. Although the pocket alarms were widely accepted and used by participants in this study, the alarm devices did not address psychosocial barriers to good adherence such as depression and stigma, which may be better handled through counseling in this population. External reminders that only inform patients when to take their pills may be less effective than those electronic devices, such as cell phone text messages, which can emulate and reinforce adherence counseling by supporting the relationship between patient and provider through interactive feedback. In this way, the positive impact of cell phone text messaging on adherence as recently demonstrated by Lester et al. may be interpreted as less a function of the cell phone as a reminder device but rather as a means to provide regular patient support [47]. The failure of alarm devices to demonstrate any impact on adherence or virologic outcomes suggests that contrary to previous assumptions [48], reminding patients when to take their medication may not be the primary constraint to adherence in sub-Saharan Africa.

This study enrolled 400 participants and followed them for 18 mo, making it one of the largest and longest, single-site, randomized trials on adherence interventions and biological outcomes [21],[22],[49]. Two distinct and important interventions were assessed concurrently in a factorial trial, allowing parallel comparison of interactive counseling and more passive alarm-reminder interventions as adherence interventions. Multiple outcomes were regularly measured including manual monthly pill counts, HIV-1 plasma viral levels, CD4 counts, and mortality. Participants were antiretroviral naïve and initiated the same free HAART regimen: a single fixed-dose combination pill (d4T, 3TC, nevirapine) taken twice a day. Based in a semi-private mission hospital in Nairobi and located near Kibera, one of the largest slums in Africa, the study clinic drew patients from a wide variety of socioeconomic and educational backgrounds [50]. Adherence among all study participants were comparable to the generally high levels found throughout sub-Saharan Africa [4], with 91% having adherence ≥80% over 18 mo. These study strengths support the validity and broad applicability of adherence counseling among antiretroviral treatment programs in resource-limited settings.

There are several limitations to the study. The intent-to-treat analysis was modified to include only those who initiated HAART since pill count was a necessary method of measuring adherence. In an analysis comparing those who were lost to follow-up and those who were retained, the study was biased towards retaining those who may have been slightly poorer and therefore potentially more receptive to attentive counseling and free medications. This may have resulted in associations that are more applicable to a poorer population. Despite randomization, baseline differences between intervention arms were noted; these were adjusted for in multivariable analyses if they were related to the outcome of interest [36]. Adherence in this study was measured using clinic-based monthly manual pill counts. Although there is no gold standard for measuring adherence to medications [51], pill counts may overestimate adherence since missing pills may not have been ingested by the participant but instead have been shared, discarded, or lost [52]. Similarly, use of the alarm device was measured by self-report and was not able to be verified. It is possible that continuous use of the alarm device was overestimated and that the insignificant associations found in this study may have been due to poor participant participation in this intervention. The adherence counseling intervention was not associated with decreased mortality. The lack of an association may have been due to relatively short follow-up and lack of power due to few mortality events. Given that virologic failure precedes death due to poor adherence, the demonstrated impact of adherence counseling on virologic outcomes in this study suggests that beneficial effects on mortality might have been apparent if the study was larger and had longer follow-up [53].

Definitions of viral failure and poor adherence are variable. The threshold of viral failure in this study was defined using WHO guidelines (HIV-1 RNA ≥5,000 copies/ml) [35], and an association with adherence counseling was also demonstrated at lower thresholds (HIV-1 RNA ≥1,000 copies/ml). However, the threshold of viral failure may be defined differently [54],[55]. Adequate adherence defined as taking ≥95% of prescribed pills derives from literature examining regimens containing un-boosted protease inhibitors (PIs) [11]. The impact of counseling on adherence <95% was not significant in this study. An adherence level of <80% was defined as poor adherence in this study, because HAART containing NNRTIs and not PIs were used and NNRTI-based regimens are most commonly prescribed in resource-limited settings [5],[6]. This definition is supported by study evidence showing that adherence <80% was significantly associated with HIV-1 RNA ≥5,000 copies/ml.

In summary, we found that intensive counseling on adherence to antiretroviral medications around the time of HAART initiation significantly reduced poor adherence and virologic treatment failure, while using an alarm device had no impact. Investment in careful individualized counseling at the onset of HAART appears to have sustained benefit. These findings are highly relevant to other HIV clinics caring for large numbers of patients in sub-Saharan Africa. Implementing adherence counseling or interventions that strengthen the relationship between the provider and patient through communication, education, and trust may substantially reduce the risk of antiretroviral treatment failure. Supporting the bond between the clinic and patient in this way appears to be more effective than using a device that simply reminds patients when to take their pills. Through interactive counseling and communication, fewer patients may need to switch to expensive second-line medications and the spread of resistant HIV may decrease.

## Supporting Information

Text S1 Trial protocol.
(0.33 MB PDF)

Text S2 CONSORT checklist.
(0.22 MB DOC)

## Abbreviations

CI: confidence interval
HAART: highly active antiretroviral therapy
HR: hazard ratio
NNRTI: non-nucleoside reverse transcriptase inhibitor
