# Supplementary material for: A Randomized Controlled Trial Comparing the Effects of Counseling and Alarm Device on HAART Adherence and Virologic Outcomes
Source: PLoS Med. 2011 Mar 1;8(3):e1000422. doi: 10.1371/journal.pmed.1000422 (PMC3046986; doi:10.1371/journal.pmed.1000422)
Supplement: Text S1 — Trial protocol. (0.33 MB PDF) [file pmed.1000422.s001.pdf]

Randomized Control Trial of Adherence Interventions

PROCEDURE MANUAL
May 2006

Table of Contents

CHAPTER 1 -- Personnel
Organogram for ARV Study 2006-2007……………………………………..…...……1
Duties and Responsibilities…………………………………………………..……...2
Study Nurse Coordinator………………………………………………………...2
Administrator……………………………………………………………………4
Data Manager……………………………………………………………………5
Data Clerk………………………………………………………………………..6
Pharmacy Technologist…………………………………………………………..7
Lab Technologist…………………………………………………………………8
Lab Assistant……………………………………………………………………..9
Community health workers……………………………………………………...10

CHAPTER 2 -- Randomization Protocol
Arm A (Adherence Counseling)…………………………………………………………12
Arm B (Alarm Device) …………………………………………………………………14
Arm C (Adherence Counseling and Alarm Device) ………………………………………16
Arm D (Control) ………………………………………………………………………...19

CHAPTER 3 -- Laboratory
Laboratory Protocols ……………………………………………...……………..20
Specimen Collection ……………………………………………………………….22

CHAPTER 4 -- Clinic Flow
Recruitment ………………………………………………………………………...23
Enrollment …………………………………………………………………………..25
Appointments Prior to Initiating HAART……………………………………………28
Medical Appointments and Follow-up………………………………………………..29
Pharmacy Appointments and Follow-up……………………………………………...30
General Counseling……………………………………………………………………31
Loss to Follow-up……………………………………………………………………..32

CHAPTER 5 -- Data Management
Databases……………………………………………………………………………...33
Facilities……………………………………………………………………………….35
Adherence Research Questionnaires…………………………………………………..36
Hope Center Questionnaires…………………………………………………………..39
Pharmacy Database……………………………………………………………………40
Safety, Security, Storage……………………………………………………………….42
Data Schedule…………………………………………………………………………43
Sample Data Work Schedule…………………………………………………………..46

CHAPTER 6 -- Pharmacy
Pharmacy Protocol……………………………………………………………………..47

CHAPTER 7 -- Recruitment

Pre-Screening Talking Points for Clinical Staff……………………………………50
Talking Points for Hope Center Staff………………………………………………51
Talking Points for Recruitment……………………………………………………..53

CHAPTER 8 -- Counseling Education and Interventions

Guidelines for Adherence Counseling……………………………………………...55
Guidelines for Pharmacist…………………………………………………………..63

CHAPTER 9 -- Questionnaires

Enrollment …………………………………………………………………………65
Address and Intake …………………………………………………………………67
Follow-up …………………………………………………………………………..69
Home Assessment …………………………………………………………………71
Home Tracing ……………………………………………………………………...73
Knowledge Assessment ……………………………………………………………74
Loss to Follow-up …………………………………………………………………76
Mortality …………………………………………………………………………...77
Adherence Counseling #1 …………………………………………………………79
Adherence counseling #2 …………………………………………………………..80
Adherence counseling #3 …………………………………………………………..81

CHAPTER 10 -- Questionnaire Definitions

General Information ………………………………………………………………..82
Enrollment Form …………………………………………………………………...84
Address and Intake Form …………………………………………………………..89
Home Assessment Form …………………………………………………………...92
Follow-up Form ……………………………………………………………………94
Home Tracing Form ………………………………………………………………..96
Knowledge Assessment Form ……………………………………………………...97
Loss To Follow-up Form …………………………………………………………..98
Mortality Form ……………………………………………………………………..99

CHAPTER 11 -- Tracking Forms

Study Calendar Template …………………………………………………………100
Pharmacy Follow-up ……………………………………………………………...101
Arm A (Adherence Counseling) …………………………………………………103
Arm B (Alarm Device) …………………………………………………………..104
Arm C (Adherence Counseling & Alarm Device) ………………………………..105
Arm D (Control) …………………………………………………………………106

CHAPTER 12 -- Consent Form

Written Consent Form ……………………………………………………………..107

CHAPTER 13 -- Data Safety and Monitoring

Data Safety and Monitoring Plan …………………………………………………111

ORGANOGRAM FOR ADHERENCE STUDY 2006-2007

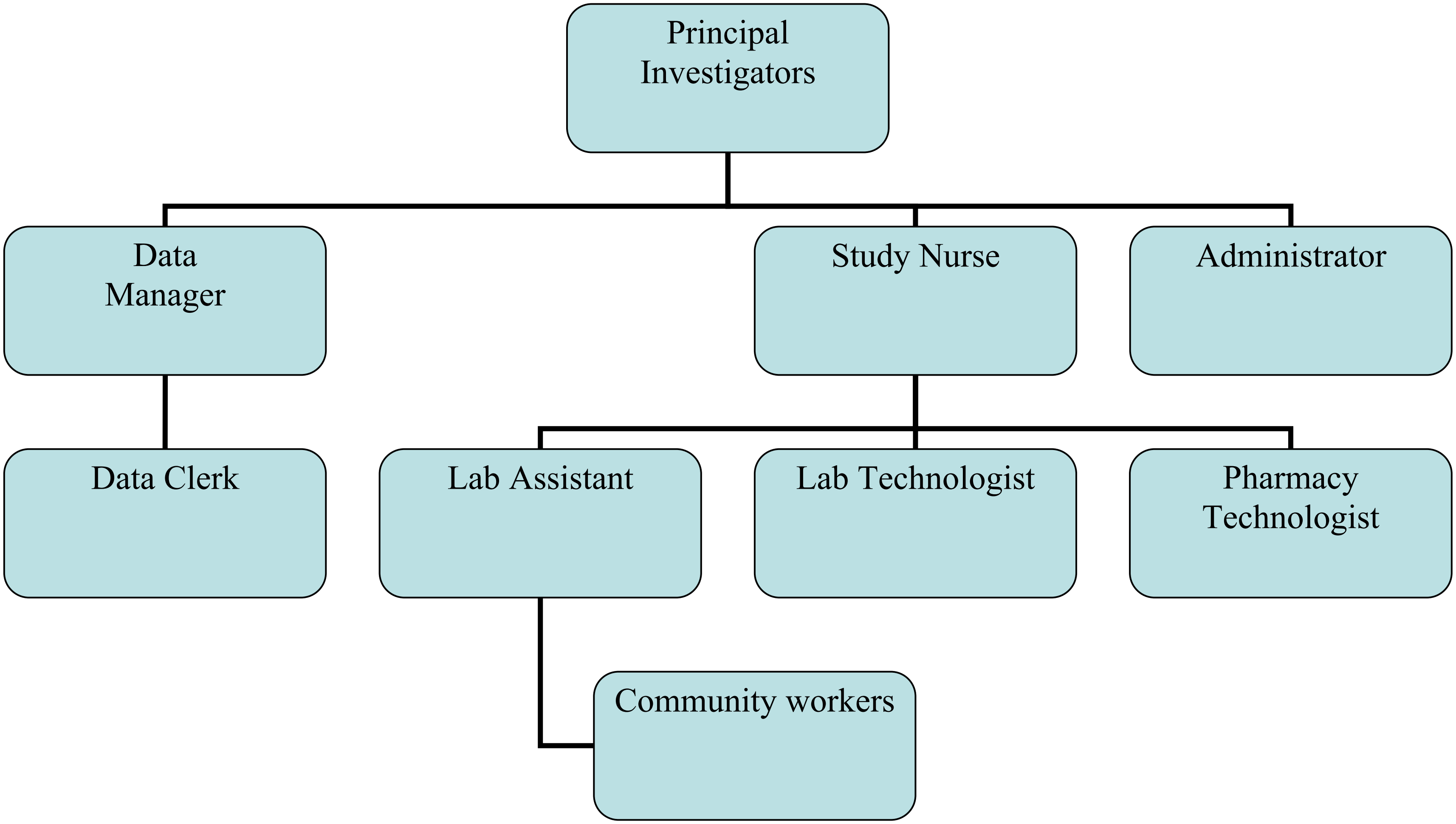

Duties and Responsibilities

Study Nurse Coordinator

- Work with Coptic Hope Center staff to ensure high recruitment for research study
- Analyze recruitment rates, determine if rates match expectations, and implement plans that will promote recruitment
- Review and confirm eligibility of each patient for research study
- Check age, mental competency, CD4 count, viral load, clinical stage, HIV-naïve status for every subject eligible for the study
- Provide adequate knowledge and education to patient so they can sign an Informed Consent
- Administer the Informed Consent and store it safely
- Enroll, randomize, and ensure follow-up of study participants
- Follow subjects enrolled in the study and ensure they remain in the study are properly followed in both the research side and clinic sides
- Help monitor research files and ensure the appropriate completion of tasks on the data entry sheets
- Determine if any patients are missing or lost to follow-up on a monthly basis and take action to report and bring these patients back under care and supervision
- Supervise community workers in the recruitment and follow-up of subjects in the study
- Oversee any transportation budget given to the community workers
- Ensure that subjects remain in their randomized arm of the study and receive the proper intervention
- Ensure that Hope Center counselors are properly administering adherence counseling interventions

- Maintain regular contact with the Hope Center clinical officers to ensure that eligible patients are being informed of the study and are being properly referred for recruitment and enrollment
- Oversee and ensure that patients in the study are receiving good medical HIV care and inform the Principal Investigator of any complications
- Every Thursday, check that all patients are receiving medications and coming are scheduled for appropriate medical, pharmacy and counseling appointments
- Prepare new patient charts
- Meet with the Data Manager to ensure that charts are prepared and entered adequately
- Ensure that patient charts are properly filled, documented, and stored
- Administer questionnaires
- Ensure completed questionnaires are delivered to the data clerk within 24 hrs
- Present weekly and monthly reports of clinic activities
- Guide and supervise clinical, research, and counseling activities
- Counsel patients as needed
- Supervise and oversee the work of the laboratory technologist, pharmacy technologist, laboratory assistant, and community workers
- Supervise the data clerk along with the Data Manager
- Maintain a diary of visits by clients to track their progress and clinic attendance
- Report on the missed appointments of study subjects using the study appointment diary and pharmacy appointment diary
- Keep track of missed appointments
- Setup the Alarm device intervention for subjects as needed
- Ensure alarm devices are functioning appropriately
- Monitor the quality of integrity of Adherence Counseling sessions

- Help collect laboratory specimens from study participants as needed
- Identify subjects that require medical attention and refer them for care at the Hope Center
- Provide senior administrative support for the research study
- Record and manage clinical attendance by research clinic staff
- Liaison with Coptic Hope Center for Infectious Diseases and provide information to Hope Center administrators as requested
- Coordinate and initiate activities that improve the conduct of the study
- Present weekly summaries along with the Study Coordinator marking progress in enrollment and tracking of subjects in the study for the Friday Administrative meetings
- Perform any other duties and responsibilities that may be given by the Principal Investigator

Administrator

- Reconcile the receipts to send to Seattle
- Communicate with Seattle when more funds are needed
- Be a liaison with the bank
- Manage study budgets
- Manage petty case
- Prepare and pay salaries
- Make purchases and keep inventories of supplies
- Maintain communication between the clinic and office
- Manage and arrange transportation
- Maintain files of personnel, correspondence, applications, receipts, budget, inventories, etc…

- Attend and take minutes at weekly administrative meeting and present them at the next meeting
- Coordinate staff evaluation procedures
- Oversee and record the attendance of office and clinic staff
- Make monthly reports on the administration of the study
- Make monthly reports of project expenses

Data Manager

- Oversee the work of the data clerk as below and assume any of the duties of the clerk that may be required due to his absence or inability to perform
- Coordinate data information entered by the data clerk on study patients with Coptic Hope Center for Infectious Disease
- Coordinate the implementation, use, and cleanliness of the pharmacy database with the pharmacist technologist and the data clerk
- Coordinate the transfer of data with the Coptic Hope data manager to the research databases with the data clerk
- Maintain daily, weekly, and monthly work schedules for the data clerk and ensure their completions
- Conduct weekly data quality checks
- Thoroughly clean the data every 3 months to ensure cleansing of errors
- Sort out any data entry or error problems weekly with the Study Nurse and Principal Investigators
- Run frequencies and range checks to identify extreme values monthly
- Coordinate the data-checking process and produce a report on the data quality
- Present weekly and monthly reports of data analysis

- Prepare monthly summary tables of numbers on number of people enrolled, pill count and self-reported adherence, and trends of cd4, weights, viral load over time
- Present weekly summaries along with the Study Coordinator marking progress in enrollment and tracking of subjects in the study for the Friday Administrative meetings
- Perform other duties that may be given by the Investigators

Data Clerk

- Enter questionnaire data and laboratory testing information into a computer database
- Create and design questionnaires and data entry forms
- Set up and maintain Access and SPSS computer databases for the research study
- Coordinate, transfer and clean medical data collected from study patients at the Hope Center for Infectious Diseases
- Manage and coordinate the research pharmacy database and ensure its accuracy and cleanliness
- Manage, coordinate, and enter GPS information into a GIS database
- Maintain and build GIS maps based on GPS information and Census data information
- Enter Census data information into GIS representations and conduct spatial analyses
- Maintain daily, weekly, and monthly work schedules and ensure their completion
- Conduct weekly data quality checks
- Check for errors in the data, correct the errors, and maintain cleanliness of the data
- Inform the Study Coordinator and Principal Investigator of any data entry problems on a weekly basis
- Run frequencies and range checks to identify extreme values monthly
- Keep a data log book of data entry queries and inconsistencies
- Coordinate the data-checking process and produce a report on the data quality

- Present weekly and monthly reports of data analysis
- Prepare monthly summary tables of numbers of people enrolled, pill count and self-reported adherence, and trends of CD4, weights, viral load over time
- Back-up all data weekly (Friday)
- Back-up all data to an off-site diskette weekly (Friday)
- Scan, verify, and check data
- Coordinate the movement of questionnaires, data forms, and information between the Hope Center clinic, the Coptic research wing, and the KNH data office
- Ensure that the computers, printers and scanner are in good order and free from viruses
- Perform any other duties that may be given by the Study Coordinator, Data Manager, and Principal Investigator

Pharmacy Technologist

- Dispense medications to patients in a timely, efficient, and correct manner
- Deliver pharmacy and adherence counseling and advice
- Monitor and ensure the completion of knowledge assessments and appropriate times of specimen collection
- Educate patients about side effects of medications and what to do if severe side effects occur
- Determine if patients on medications require further medical attention due to drug side effects or medical conditions
- Administer questionnaires and enter records
- Record pill count, electronic pill counts, and missed appointments on each patient
- Maintain accurate database and inventory of medications dispensed as well as clinical and adherence measurements

- Coordinate work and dispensation of medications with the Hope Center pharmacy and their inventory
- Follow patients closely who are in the study and determine if any are lost to follow-up or non-adherent on daily basis
- Inform Community workers and Study Coordinator if any patients miss their appointments or are lost-to-follow-up
- Ensure that patients pick up their antiretroviral mediations from the pharmacy monthly
- Dispense and distribute medications associated with opportunistic infections
- Confer and communicate with Hope Center clinicians if any questions or problems arise concerning the prescription, medication side effects, or other medically related issues
- Prepare a weekly report for the Study Coordinator of patients picking up their medications at the pharmacy and their measured levels of adherence
- Develop and update the pharmacy database system as needed
- Ensure that the supply of medications required for the study is available and not out of stock at any time
- Assist the Study Coordinator in the recruitment of study participants
- Perform any other duties that may be given by the Study Coordinator or Project Investigator

Laboratory Technologist

- Manage the laboratory at the Coptic Hope Research Center and ensure equipment and supplies are available
- Collect laboratory specimens from the study participants
- Conduct laboratory tests on specimens that are collected

- Ensure the quality of the laboratory facilities
- Ensure the high quality of lab results
- Supervise the laboratory and lab assistant
- Separate, freeze, and process specimens at KNH or Coptic
- Oversee and ensure that lab specimens at KNH or Coptic are properly separated and frozen.
- Give results of test in a timely and efficient manner
- Prepare the shipment of lab specimens to Seattle from Coptic Hospital and KNH lab in Nairobi
- Manage and keep track of samples that are collected and sent to Seattle
- Work in clinic on weekends as needed to separate and freeze specimens
- Present weekly and monthly reports of laboratory activities
- Perform other duties that may be given by the Study Nurse or Investigators

Laboratory Assistant

- Collect laboratory specimens from study participants
- Keep track of laboratory specimens
- Conduct laboratory tests on specimens that are collected
- Give results of tests in a timely and efficient manner
- Ensure the high quality of lab results
- Setup the Alarm device intervention for subjects and dispense the alarm device
- Ensure alarm devices are functioning appropriately
- Manage and keep track of study patients appointments at Hope Clinic
- Perform home assessments, mapping, and home visits as necessary
- Supervise and help manage Community workers
- Administer Knowledge Assessment questionnaires

- Review all patient charts to ensure proper randomization before study patient visits pharmacist
- Ensure timely transportation of specimens to KNH on a daily basis
- Separate, freeze, and process specimens at KNH or Coptic as needed
- Prepare the shipment of lab specimens to Seattle from Nairobi
- Work in clinic on weekends as needed to separate and freeze specimens
- Ensure timely transportation of questionnaires, research files, and data flash disk between Coptic and KNH offices
- Locking up GPS devices every evening
- Perform other duties that may be given by the Study Coordinator or Principal Investigator

Community health workers (CHW)

- Conduct home assessments on all study participants
- Map and record the location of homes of participants
- Do home visits to follow-up participants missing in clinic
- Record the time and route needed to attend the subjects home
- Fill out study questionnaires on household information on study participants
- Keep track of participants appointments in a personal diary book
- Update the study diary with patient appointments
- Sign and date the completion of subject appointments in the study diary and research file
- Determine whether subjects have missed clinic appointments and inform the Study Coordinator if appointments are missed
- Follow-up subjects who miss appointments as per the Study Coordinator's requests
- Clean and help organize study clinic

- Perform duties given by Study Coordinator and Lab Assistant
- Perform other duties that may be given by the Investigators

Arm A (Adherence Counseling)

Subjects randomized to Arm A will receive 3 sessions of educational adherence counseling. Two sessions will be completed before initiation of antiretroviral medications and one session will occur one month after. The first session will be an education session lasting for approximately 45 minutes and will cover topics including the cause of AIDS, the purpose of obtaining CD4 counts, the problems of drug side effects, and the dangers of viral resistance. A copy of the educational counseling protocol used in this first session is included in this chapter. The second session will assess the knowledge gained by the subject from the first session. If the subject fails in this assessment, either through poor knowledge, lack of demonstrated commitment, or community worker's judgment, then adherence session one will be repeated as many times as necessary. If the subject passes the second session then they may initiate antiretroviral medications that will be dispensed by the Study Pharmacist.

Upon randomization to Arm A, the study coordinator will insert the HAART Protocol sheet specific for Arms A and C in the subject's Hope medical folder. On the HAART Protocol sheet, adherence sessions will be marked as needing completion before the HAART Protocol is approved. After specimens are drawn in the laboratory, the Coordinator will send the subject to the Hope Center reception with a community worker who will schedule the subject for the adherence counseling sessions. After determining the dates from the receptionist, the community worker will log the counseling appointments in her calendar book, the subject's research file, and the research calendar book.

A research calendar book will be kept that tracks all subjects and shows which subjects are returning on each day so that the coordinator and community worker can determine if there are missed appointments. Subjects will be asked to stop by the research office on their way to clinic to log their clinic attendance in the large research calendar book. The research calendar book will be overseen by the study coordinator and maintained by the

Laboratory Assistant. Subjects will be asked and required to schedule clinic appointments at the Hope reception with a community worker present who can record these dates.

The community worker will track the subjects and their attendance in the counseling sessions. They will confirm that the subjects have attended all the counseling sessions prior to picking up antiretroviral medications from the study pharmacist. The study pharmacist will check the tracking form and ensure that adherence counseling sessions one and two and other study protocols have been completed prior to HAART initiation. At antiretroviral initiation, the community worker will check to ensure that the third adherence session is scheduled, and will note these appointments in the calendar books as above. At the Month 1 appointment with the pharmacist, the study pharmacist will check the tracking form if the subject has completed adherence session three. If this has not been done, the community workers and coordinator will be informed and this will be scheduled and completed before Month 2 pickup of antiretroviral medications from the study pharmacist.

If any adherence counseling appointments are missed, the community worker will note this through her calendar book and begin tracking the subject through phone calls and home visits with the knowledge and approval of the study coordinator.

Arm B (Alarm Device)

Subjects randomized to Arm B will receive an alarm device along and NO sessions of educational adherence counseling. Alarm devices will be programmed by the laboratory assistant to signal twice a day at a time most convenient to the subject. These pocket alarms devices will be carried at all times by the subjects and can be viewed as a pocket watch. Six months after initiating therapy and carrying the alarm device, the device will be brought back to the laboratory assistant who will disarm the alarm but continue to allow the subjects to carry the alarm as a pocket watch.

Upon randomization to Arm B, the study coordinator will insert the HAART Protocol sheet specific to Arms B and D in the subject's Hope medical folder. On the HAART Protocol sheet, NO adherence counseling sessions will be scheduled and adherence counseling will not be necessary for HAART Protocol approval and completion. After specimens are drawn in the laboratory, the coordinator will send the subject to the Hope Center reception with a community worker who will schedule the subject for the HAART Protocol. The community worker will ensure that the adherence counseling sessions will NOT be performed for any of these subjects in Arm B. Community workers will ensure that the clinic receptionist only books subjects for Social Work and Nutritionist visits. After determining the dates for these visits from the receptionist, the community worker will log the counseling appointments in her calendar book, the subject's research file, and the research calendar book.

A research calendar book will be kept that tracks all subjects and shows which subjects are returning on each day so that the coordinator and community worker can determine if there are missed appointments. Subjects will be asked to stop by the

research office on their way to clinic to log their clinic attendance in the large research calendar book. The research calendar book will be overseen by the study coordinator and maintained by the laboratory assistant. Subjects will be asked and required to schedule clinic appointments at the Hope reception with a community worker present who can record these dates.

At antiretroviral initiation, the study pharmacist will check the tracking form and ensure that the subject receives an alarm device from the laboratory assistant before receiving medications. Once the subject has obtained the programmed device from the laboratory assistant, the pharmacist will dispense HAART and follow the subject monthly. Any difficulties regarding the alarm device will be handled by the laboratory assistant. Lost or stolen alarm devices should be reported to the study investigators immediately.

At the Month 6 visit at the pharmacy for medication pick-up, the study pharmacist will check the tracking form and determine that the alarm device is to be returned to the laboratory assistant to be deactivated. At this visit, the alarm function of the device will be turned off. Thereafter, if requested, the device can be returned to the subject for use as a pocket watch. The study register/calendar will be checked regularly by the community workers, pharmacist, laboratory assistant, and coordinator to ensure that these devices are returned or switched off 6 – 7 months after HAART initiation.

Arm C (Adherence Counseling and Alarm Device)

Subjects randomized to Arm C will receive 3 sessions of educational adherence counseling and an alarm device. Two adherence sessions will be completed before initiation of antiretroviral medications and one session will occur one month after. The first session will be an education session lasting for approximately 45 minutes and will cover topics including the cause of AIDS, the purpose of obtaining CD4 counts, the problems of drug side effects, and the dangers of viral resistance. A copy of the educational counseling protocol used in this first session is included in this chapter. The second session will assess the knowledge gained by the subject from the first session. If the subject fails in this assessment, either through poor knowledge, lack of demonstrated commitment, or community worker's judgment, then adherence session one will be repeated as many times as necessary. If the subject passes the second session then they may initiate antiretroviral medications that will be dispensed by the Study Pharmacist.

Alarm devices will also be given to subjects and be programmed by the laboratory assistant to signal twice a day at a time most convenient to the subject. These pocket alarms devices will be carried at all times by the subjects and can be viewed as a pocket watch. Six months after initiating therapy and carrying the alarm device, the device will be brought back to the laboratory assistant who will disarm the alarm but continue to allow the subjects to carry the alarm as a pocket watch as they desire.

Upon randomization to Arm C, the study coordinator will insert the HAART Protocol sheet specific for Arms A and C in the subject's Hope medical folder. On the HAART Protocol sheet, adherence sessions will be marked as needing completion before the HAART Protocol is approved. After specimens are drawn in the laboratory, the Coordinator will send the subject to the Hope Center reception with a community worker who will schedule the subject for the adherence counseling sessions. After determining the

dates from the receptionist, the community worker will log the counseling appointments in her calendar book, the subject's research file, and the research calendar book.

A research calendar book will be kept that tracks all subjects and shows which subjects are returning on each day so that the coordinator and community worker can determine if there are missed appointments. Subjects will be asked to stop by the research office on their way to clinic to log their clinic attendance in the large research calendar book. The research calendar book will be overseen by the study coordinator and maintained by the Laboratory Assistant. Subjects will be asked and required to schedule clinic appointments at the Hope reception with a community worker present who can record these dates.

The community worker will track the subjects and their attendance in the counseling sessions. They will confirm that the subjects have attended all the counseling sessions prior to picking up antiretroviral medications from the study pharmacist. The study pharmacist will check the tracking form and ensure that the alarm device, adherence counseling sessions one and two and other study protocols have been completed prior to HAART initiation. The alarm device will be handed out by the laboratory assistance. At antiretroviral initiation, the community worker will also check to ensure that the third adherence session is scheduled, and will note these appointments in the calendar books as above. At the Month 1 appointment with the pharmacist, the study pharmacist will check the tracking form if the subject has completed adherence session three. If this has not been done, the community workers and coordinator will be informed and this will be scheduled and completed before Month 2 pickup of antiretroviral medications from the study pharmacist.

If any adherence counseling appointments are missed, the community worker will note this through her calendar book and begin tracking the subject through phone calls and home visits with the knowledge and approval of the study coordinator. Any difficulties

regarding the alarm device will be handled by the laboratory assistant. Lost or stolen alarm devices should be reported to the study investigators immediately.

At the Month 6 visit at the pharmacy for medication pick-up, the study pharmacist will check the tracking form and determine that the alarm device is to be returned to the laboratory assistant to be deactivated. At this visit, the alarm function of the device will be turned off. Thereafter, if requested, the device can be returned to the subject for use as a pocket watch. The study register/calendar will be checked regularly by the community workers, pharmacist, laboratory assistant, and coordinator to ensure that these devices are returned or switched off 6 – 7 months after HAART initiation.

Arm D (Control)

Subjects randomized to Arm D will receive NO alarm device and NO sessions of educational adherence counseling. Upon randomization to Arm B, the study coordinator will insert the HAART Protocol sheet specific to Arms B and D in the subject's Hope medical folder. On the HAART Protocol sheet, NO adherence counseling sessions will be scheduled and adherence counseling will not be necessary for HAART Protocol approval and completion. After specimens are drawn in the laboratory, the coordinator will send the subject to the Hope Center reception with a community worker who will schedule the subject for the HAART Protocol. The community worker will ensure that the adherence counseling sessions will NOT be performed for any of these subjects in Arm B. Community workers will ensure that the clinic receptionist only books subjects for Social Work and Nutritionist visits. After determining the dates for these visits from the receptionist, the community worker will log the counseling appointments in her calendar book, the subject's research file, and the research calendar book.

A research calendar book will be kept that tracks all subjects and shows which subjects are returning on each day so that the coordinator and community worker can determine if there are missed appointments. Subjects will be asked to stop by the research office on their way to clinic to log their clinic attendance in the large research calendar book. The research calendar book will be overseen by the study coordinator and maintained by the laboratory assistant. Subjects will be asked and required to schedule clinic appointments at the Hope reception with a community worker present who can record these dates.

Laboratory Protocols

PROCEDURE FOR ISOLATION OF PLASMA

1. **Collection and transportation of Samples from RCT Adherence Intervention Clinic**
    - Lab Assistant will collect one tube of blood sample in a well-labeled 10 ml tube (purple top)
    - After collection of sample details will be recorded in clinic lab book**.** Details include:
        - Specimen ID
        - Date
        - Visit (enrollment, month 6, ect)
        - Time sample was collected
    - Samples should be placed in a cool box and transported to laboratory at KNH before 4 pm or to the Coptic laboratory.
2. **Receiving Samples**
    - All details of sample delivered will be recorded in a laboratory research book
        - Specimen ID, Date, number of tubes, and blood volume
3. **Preparation of Workbench at laboratory**
    - Wear an appropriate size pair of latex gloves.
    - Clean your work surface with 70% alcohol or > 10% Jik diluted in tap water.
    - Prepare work area in the hood; bleach bucket, racks, tips, gauze, ect.
4. **Plasma Separation**
    - Spin blood at 1,800 rpm for 10 minutes at room temperature and set brake to low (make sure to put a cap on the bucket during the spin). Record all spins in centrifuge logbook.

- Label 2 serum/plasma vials for each patient with Specimen ID, Specimen part, and Date
- Draw off 2ml of plasma from spun cells and place in well labeled serum vial
- Inventory serum vials in logbook

5. Plasma Sample Storage

- Place the samples into a box designated for storage of plasma in the -80°C freezer, record the location in the lab storage book, and enter details in the lab book and computer database.

Specimen Collection

Time Point	Type	#	Tube	Tests	Where
Enrollment	Whole Blood	1	Large purple	Plasma Separation	KNH/Coptic
Month 6	Whole Blood	1	Large purple	Plasma Separation	KNH/Coptic
Month 12	Whole Blood	1	Large purple	Plasma Separation	KNH/Coptic
Month 18	Whole Blood	1	Large purple	Plasma Separation	KNH/Coptic

Research Clinic Flow

Recruitment

Participants for the HAART Adherence study will be recruited from the Coptic Hope Center for Infectious Disease. Participants recruited for this study will have to meet the following eligibility criteria:

1) HIV-positive and eligible to start HAART
2) Age (18 years of age or older)
3) Willing to begin HAART
4) HAART treatment-naïve
5) Agree to home visits

Participants for the study will be excluded if they are pregnant, under the age of 18, or are mentally disabled whether through AIDS or another disease and are unable to start a fixed-dose drug regimen.

Medical and clinical officers at the Hope Center will help identify patients that meet eligibility criteria for this study. Once a medical professional has identified these patients they will assess if the patient is willing to learn more about the research study. If the patient is not interested in the study, the patient will continue receiving services at Hope Center. Each medical officer will address interest by giving a brief statement about the study. For more information on talking points for recruitment please review chapter seven.

If the patient is interested in learning more about the research study, the clinical officer will phone the research facility and speak with the study coordinator. The community health worker will carry the patient's Hope Center file and guide the patient to research facility. The participant will be introduced to the study coordinator who will explain more about the study and enroll the patient if they are interested in participating.

Overview of Study Recruitment

The study nurse will introduce the purpose of the research study. In addition she will review the risks and benefits of being involved in participating in the research study. She will also review and confirm that the patient is eligible for the study.

If the subject continues to be interested in participating in the research study and meets eligibility criteria, the patient can be enrolled into the Adherence Study.

Enrollment

- Informed Consent

The subject will be required to understand and sign this document. The study coordinator will give the patient the consent in Kiswahili or English to read. If the participant has any questions the study coordinator shall answer all questions prior to enrollment. If the participant agrees verbally and signs the written consent, the patient will be enrolled into the research study.

- Randomization

The study coordinator will start by assigning the subject a research ID number. She will keep track of all research ID numbers in her log book. After assigning the subject with an ID number, she will select the study envelop corresponding to that research ID number which will randomly place the subject in one of four arms.

- Transferring of Information – Creating a new Hope File and research file

The study coordinator will then move all the information from the Hope file to a new Hope file (which will be a different color from previous Hope File). She will also place a HAART protocol checklist which will assign the appropriate interventions for the subject depending on which arm of the study the participant has been placed. In addition the study coordinator will exchange the appointment card given at the Hope Center with a different color appointment card. She will write the research ID number in the right hand corner of the appointment card and will write the arm the subject has been placed in left-hand corner.

The randomization envelope will then be placed in a new Adherence Study Research file that will be used to keep track of all research information. The file will be

selected based on which Arm the patient was selected for. The study coordinator will place the research ID number on the front of the file. In addition place the signed consent into the research file. The study coordinator will then administer 2 questionnaires: Address and Intake and Enrollment.

- Laboratory Specimen Collection/Knowledge Assessment

After the subject has completed these questionnaires, the participant will be taken to the laboratory. Here the lab assistant will introduce himself and administer the Knowledge Assessment questionnaire. He will place the questionnaire within the research file which will kept in the laboratory. After this the lab assistant will begin procedures for specimen collection.

- Appointment Schedule

After specimen collection the new Hope Center file, appointment card, and the subject will be assigned a community health worker. This CHW will accompany the subject to the Hope Center reception to help schedule visits according to the patient's HAART Protocol checklist. The appointments that will be booked for each group are below:

Arm A and C: Book 4 appointments for the HAART protocol

i. Week 1: Adherence 1 and Social Worker

ii. Week 2: Adherence 2 and Nutritionist

Arm B and D: Book 1 appointment for the HAART protocol

iii. Week 1: Social Worker and Nutritionist

- Recording Appointments

The CHW will record the dates for these appointments on the subject's appointment card and their own study diary. After this they return back to the study laboratory to record the appointments in the Adherence study research file and the study schedule binder.

- Home Assessment

If there is time, the CHW will accompany the patient home to complete the Home Assessment questionnaire. If this is not possible the CHW will schedule a home visit with the patient at the next appointment. This assessment must be completed prior to the start of antiretroviral medication. The assessment will include the collection of GPS coordinates of the subject's home. The GPS device will be given by to the CHW by the laboratory assistant. Transportation funding will be given by the Study coordinator; money will be given for one-way transportation of the subject and two-way trip for the CHW. Petty cash will be kept by the study coordinator and a record book will be used to keep track of all funding.

Appointments Prior to Initiating HAART

Subjects will be required to schedule appointments at the Hope Center and complete the HAART protocol according which group (arm) they have been placed in. All appointments will be made with the help of the community health workers. The Hope Center reception will be informed that all appointments for research subjects must be accompanied by a CHW.

Subjects should present themselves to the research study clinic prior to there Hope Center appointments. They will check-in with the CHWs or study coordinator who will sign the study calendar that the patient has arrived for their appointment.

For every appointment the subject is advised to stop by the study office to check-in prior to Hope visit. After Hope visit, the subject will inform the study office to help make appointment with community health worker.

After each subject has finished the HAART protocol, according to the arm they were placed in, they will accompany the community worker to help book an appointment to see the medical/clinical officer. The medical professional, will make the decision to start antiretroviral medications.

If the subject is approved to start antiretroviral medications, his or her file must be brought to senior Hope management to certify completion of the HAART protocol. After certification, the participant will accompany the community health worker to the reception to book appointment for medical visits after two weeks and adherence 3 in six weeks (if the participant is assigned to adherence counseling). Then the subject will be referred to pick-up medications at the study pharmacy.

Medical Appointments and Follow-up

After starting medications, the patient will visit the medical officer after two weeks to review medication side effects. If subject is tolerating medications, the community health worker will help the subject book an appointment to see the clinical officer in 2-3 months for further follow-up.

All medical concerns will be within the purview of the Hope Center for Infectious Disease. The subject will be seen every 2-3 months for medical follow-up throughout the course of the study. Every six months a CD4 count and viral load will be conducted. This information will be gathered via the medical follow-up and lab forms scanned into the Hope Center database. All medical visits will be recorded and followed by the community health workers.

If the subject becomes ill, or needs medical attention, they can be seen at the Hope Center at any point during the study. The study coordinator will request that the subject stop by before to inform the study that he/she is in need of medical attention. These appointments will be recorded as Medical PRN visits in the study calendar.

Pharmacy Appointments and Follow-up

The study pharmacy technologist will review the patient's research file to verify completion of adherence interventions. If the patient is to receive the alarm device, she will refer the patient to see the lab assistant or the study nurse to receive the study device. After which she will give the patient counseling on the use and side-effects of antiretroviral medications. She will book the participant to see her in two weeks to increase the medications. She will record the scheduled visits in the study file and pharmacy calendar file. In addition this appointment will be kept in the computer pharmacy database.

When the subject returns, the pharmacy technologist will perform a pill count and a follow-up questionnaire form. After which the pharmacist will book an appointment to see the subject every 4 weeks.

At the end of month one, the pharmacist will verify that the patient has completed adherence #3 (Arm A and Arm C), is using the alarm device (Arm B and Arm C), or using neither intervention (Arm D). At the end of the 6 month visit the pharmacist will make sure that the alarm device will be stopped and retrieved by the study pharmacist (for arms B and D). In addition the pharmacy technologist will ensure the completions of appropriate knowledge assessment questionnaires and collection of lab specimens at month 6, month 12, month 18. For more information on the pharmacy please review chapter six.

General Counseling

At any time the subject can be recommended to receive more counseling in regards to social issues; this includes problems with disclosure, stigma, or economic. However the role of the counselor during these sessions will not include the adherence education and counseling which will be limited for certain arms of the study.

Loss to Follow-up

If the subject misses any of the scheduled and recorded appointments, the subject will be identified and recorded by the study coordinator. At each weekly meeting subjects who have missed appointments will be discussed. If the patient is unable to be reached, a community health worker will be recruited find the subject and file a Home Tracing form.

If the participant is not found, the subject will be discussed and a Lost to Follow-up form will be filed and placed in the subject's research file. If the subject is found to have passed away, the study coordinator and principal investigator should be notified. In addition a Mortality form will file by the study coordinator. If the patient is found, he or she will be asked a few questions and will be encouraged to return to clinic. If the subject refuses to return, the community health worker will exit the patient from the study. If the patient is found to be hospitalized, the study coordinator and principal investigator should be notified immediately, and the reason for hospitalization should be recorded.

Data Management

Databases

The study databases are recorded in Microsoft Access, SPSS, GIS software programs or a written notebook and include:

1. Questionnaires – this SPSS database includes all questionnaires that are filled at the clinic or at Coptic Hospital. In total there are 16 questionnaires:

   Scanned in at Coptic Hospital:

   - Adherence #1
   - Adherence #2
   - Adherence #3
   - Medical Screening
   - Medical Follow-up
   - Laboratory Form
   - Nutritionist Screening
   - Nutritionist Follow-up
   - Address and Intake
   - Social Worker Screening

   Scanned in at KNH:

   - Address and Intake
   - Enrollment
   - Loss to Follow-up
   - Mortality
   - Home Tracing Form
   - Home Assessment
   - Pharmacy Follow-up Form
   - Knowledge Assessment

2. Pharmacy – this database is recorded in a Microsoft Access. It keeps track of appointments, pill counts, calculate adherence and how much and which type of antiretroviral medications are being dispensed to the subject.

3. Laboratory – this data contains an inventory of all specimens collected, separated, and stored.
   - Specimen Collection – written notebooks – provide a written record that tracks where the specimens have been and where they are going.
     - Specimens include:
       - Whole Blood
     - Specimen Books:
       - Clinic Specimen Collection Book
       - Lab Specimen Collection Book
4. GIS Database -- GPS locations of subjects will be collected by community health workers using a Garmin GPS device. This data will be stored and downloaded to computers at Kenyatta National Hospital. The software used to store and analyze this data is ArcView 3.2.

Facilities

RCT Adherence Intervention Research Clinic at Coptic Hospital

The on-site facilities available for the research study include two desktop computers. One computer is devoted to programming alarm devices, while the other is designated to collect and track pharmacy information. Each computer is outfitted with a CD-burner. None of the computers have internet access.

Kenyatta National Hospital

The RCT Adherence Intervention study has a data office in the department of Obstetrics and Gynecology at the University of Nairobi, Kenyatta National Hospital. There are five desktop computers, one laser printer, one scanner, one zip drive, and two CD-burners. All computers except the data computer have internet access. All computers have antivirus software which is kept up to date by the data manager. Questionnaires are entered into the computer using Cardiff Teleform software and a Fujitsu scanner. GIS information is downloaded to the computer using ArcView 3.2 software. All computers have SPSS installed.

Hope Center at Coptic Hospital

The RCT Adherence Intervention study will bring medical data collected from the Coptic Hope Center database. At this facility there are two desktop computers, one scanner, and two CD-burners. None of these computers have internet access. All computers have SPSS installed.

Adherence Research Questionnaires

Entry

Most data is collected on study subjects using questionnaires. Each subject has a folder in which all questionnaires are stored. These folders are stored in a locked cabinet in the study clinic at Coptic Hospital research facility. Newly filled-out questionnaires are brought to the office from the clinic daily. Questionnaires are scanned into the computer database the same afternoon, and folders are returned to the clinic the following morning. Scanned data is verified by Teleform software and later cross-checked against the paper questionnaire on the same day by the data clerk after it is exported into SPSS.

Quality

1. The data clerk maintains a data logbook where all the data entry queries or errors encountered during data entry are recorded. Data entry queries or errors are then discussed with the study staff at least once a week. Discussions are held with both study staff and data clerk together at the study clinic and corrections are made to the database by the data clerk upon returning to the data office. The data manager ensures that all errors are attended to on a regular basis.
2. The data manager ensures that all the data is clean at all times. Questionnaire data is checked for entry accuracy at the time of scanning. In addition, data checking is done on the databases every week and the data manager takes responsibility for organizing the data checking process. If errors are found, the folders are brought from clinic and checked for inconsistencies. A summary report on the data quality and data entry accuracy is then produced by the data manager and distributed to the project investigators.

3. Data checking is done using the following methods:

- *Ranges and Validity rules*

  A range of acceptable values has been set for all the appropriate variables. Any values that fall outside this range cannot be accepted by the database. Validity rules are also set where certain variables can only be entered if they comply with a particular rule. For example, the database does not allow outside limit or blank entries for the patient identification numbers and all unreal dates are rejected. The data manager works with the data entry clerks to identify any inconsistent data on a weekly basis. Inconsistency checks are done when the files are in SPSS. The manager then consults with study clinicians to resolve the inconsistencies.

- *Line listings*

  The main objectives of line listings include:

  1. identifying any errors made during data entry
  2. estimating the accuracy rate of the data entry clerk, monitor and asses his/her data entry performance

  The data manager produces line listings of all the enrollment and follow-up files on a monthly basis. The data team members then check the line listings against the hard copy questionnaires. Errors are highlighted on the line listings and the error rate approximated thereafter.

  After the checking has been completed, the data manager lists all the corrections that need to be done in the patient charts on the data entry sheet,

this helps the data entry clerk easily identify the corrections that need to be done.

- *Missing values*

  The data entry clerk identifies any missing values during data entry. Where values are missing, the data entry clerk notes this down on the data entry sheet of the respective folder and in the data logbook. At least once a week, the study staff will review errors that are determined in this manner.

Hope Center Questionnaires

Entry

Entry of questionnaires of study subjects at the Hope Center is similar to entry of questionnaires at the study facility. The only exception being that each research subject has a folder (designated in a different color from other Hope patients) which is kept in a locked cabinet at Coptic Hospital Hope Center. These questionnaires are scanned into the computer database within 24 hrs and folders are returned to the clinic after scanning. Scanned data is verified by Teleform software and the data clerk keeps records of all errors made. The scanned data is exported to SPSS.

Exporting data to CD

Every Thursday, the data clerk from KNH will meet with the data manager at Coptic Hope Center. The data clerk will have a list of all Hope ID numbers for all research subjects. All the information collected about the research subject at the Hope Center in SPSS will be exported and copied to a CD which will be kept by the research data clerk. Each week new research participants' data will be added to questionnaire data

from the previous research participants. The data clerk will review information and conduct quality checks on information on a weekly basis.

Pharmacy Database

Entry

Information that is recorded in the pharmacy will be recorded using Microsoft Access. Data on pill counts and appointment schedule will be recorded directly into the database.

Exporting data to CD

Every Thursday, the data clerk from KNH will meet with the pharmacy technologist at Coptic research facility. All the information collected about the research subject will be exported and copied to a CD which will be kept by the research data clerk. The data manager and data clerk will review information and conduct quality checks on information on a weekly basis.

Quality

The quality of data collected on research subjects at the Hope Center clinic and the pharmacy database will be checked by the data clerk and data manager creating and using SPSS programs to identify errors. Three types of errors identified:

- *Missing values*

The data clerk will identify any missing values of the information collected from the exported SPSS files. Where values are missing the data clerk will note these in a log book to review with the Hope Center data manager or pharmacy technologist twice a week.

- *Range, Validity, and Inconsistency rules*

A range of acceptable values will be set for all the appropriate variables. In addition the data manager will create validity rules for certain variables which will

marked as errors if they do not comply with a particular rule. For example, the quality checking program will identify all variables outside the limit or blank entries for the patient identification numbers and all unreal dates are rejected. Violations of these rules will be marked by the data clerk and recorded in the data error log. These will be reviewed with the data manager and Coptic data manager on a weekly basis. Again the data manager will create an SPSS quality checking program which will identify problems with data. For example cross-checking variables such as the subject being male and pregnant or a greater than 50 kg increase in weight in less than a month. These will be recorded by the data clerk and discussed at weekly data meetings.

Safety

All databases are backed up every Friday by saving the most current files on two CD disks.

Security

- All computers are password-protected, preventing access by any unauthorized persons.
- Data is backed up on a weekly basis by the both the data clerks and the data manager. Data is saved on two CD disks. One set remains with the data manager and the other is kept with a study investigator at an off-site location.

Storage

All patient records are filed according to numerical order of the patients' identification. Folders are kept in a locked storage cabinet at the study clinic for reference by research personnel. Once the subject completes the study or is lost to follow-up, her records are brought to the KNH office for storage.

Data Schedule

Daily

a) Questionnaire entry – *Questionnaires* are scanned into the computer, verified, and cross-checked against the paper questionnaire at the same time. Data is exported and stored directly into SPSS. Date of data entry and name of clerk is recorded in the study subject's file.

b) Questionnaire examination – During questionnaire entry, any errors in filling out the questionnaires are noted in the *Questionnaire Error Checking Book*. Questionnaires are closely examined to ensure that all forms are properly completed. Files of subjects who have completed the study or who are lost to follow-up are stored in the office.

c) Specimen entry – Specimens that are collected in clinic are entered into the *Clinic Specimen Collection Book*. The specimens are then delivered to KNH where they are entered in the *Lab Specimen Collection Book* which is kept by the lab technologist. This method tracks the specimens.

Weekly

a) Monday

- Specimen error checking – the *Clinic Specimen Collection Book* is compared to the *Lab Specimen Collection Book* located to note any inconsistencies. This will be done by the lab technologist on a daily basis and checked once a month by the data clerk.
- Computer update – this requires defragmenting the hard drive, updating the virus library, and scanning the hard drive for viruses on all computers

b) *Tuesday*

- Questionnaire error checking – The data clerk uses the *Questionnaire Error Checking Book* to discuss any errors with study staff at the research facility and will correct all error upon returning to KNH.
- Exporting Data from Hope Center and Pharmacy databases – The data clerk will burn a CD of information collected by Hope Center on research subjects. In addition the data clerk will burn information collected on the pharmacy database.

c) *Wednesday*

- Data meeting with Coptic Data Manager – The data manager and data clerk at KNH will meet with the Hope Center Data Manager to discuss any errors within the data collected from the Hope Center questionnaires. Errors are corrected at this time.

d) *Thursday*

- Questionnaire error checking – The data clerk uses the *Questionnaire Error Checking Book* to discuss any errors with study staff at the research facility and will correct all error upon returning to KNH.
- GPS Downloading – The data clerk will bring the GPS devices from the community health workers and download the data points from previous

e) *Friday*

- Backup data – Data is backed up on two CDs. One CD and one zip disk remain with the data manager while the other set is given to a study investigator who takes it to an off-site location.
- Summary weekly– a summary and analysis of the data is generated weekly by the data manager for the Administrative meeting. This summary should include:
  - Update on number of enrolled, randomized, and lost to follow-up
  - Update on specimen collection for each subject and for whole study
  - Update on adherence data collected from the pharmacy database

Every Two Weeks

a) Inconsistency error checking – Data manager uses SPSS to check the questionnaire database for any inconsistencies. All patient charts are also brought to the office at this time to ensure that all questionnaires have been entered and the charts are in good order.

b) Line-listing error checking – Data clerk should print out a line list of the enrollment data and check them for any inconsistencies.

Week of	Monday	Tuesday	Wednesday	Thursday	Friday
**1**	Specimen Complete error check – Lab Technologist<br><br>Computer update	Questionnaire error correction at research site<br>Data clerk gets exported SPPS files for research subjects	Data mtg w/Coptic data manager	Questionnaire error correction at research site<br>GPS Downloading	Back up data<br><br>Weekly summary<br><br>Inconsistency check
**8**	Specimen Complete error check – Lab Technologist<br><br>Computer update	Questionnaire error correction at research site<br>Data clerk gets exported SPPS files for research subjects	Data mtg w/Coptic data manager	Questionnaire error correction at research site<br><br>GPS Downloading	Back up data<br><br>Weekly summary
**15**	Specimen Complete error check – Data Clerk<br><br>Computer update	Questionnaire error correction at research site<br>Data clerk gets exported SPPS files for research subjects	Data mtg w/Coptic data manager	Questionnaire error correction at research site<br><br>GPS Downloading	Back up data<br><br>Weekly summary<br><br>Inconsistency check
**22**	Specimen Complete error check – Lab Technologist<br>Computer update	Questionnaire error correction at research site<br>Data clerk gets exported SPPS files for research subjects	Data mtg w/Coptic data manager	Questionnaire error correction at research site<br><br>GPS Downloading	Back up data<br><br>Weekly summary
**29**	Specimen Complete error check – Lab Technologist<br><br>Computer update	Questionnaire error correction at research site<br>Data clerk gets exported SPPS files for research subjects	Data mtg w/Coptic data manager	Questionnaire error correction at research site<br>Line Listing of enrollment questionnaires<br>GPS Downloading	Back up data<br><br>Weekly summary<br><br>Inconsistency check

SAMPLE DATA WORK SCHEDULE

Pharmacy

Introduction

The study pharmacy is one of the most important points of contact between the research subject and the study. The study pharmacist will be seeing subjects most frequently among study staff since subjects will be picking up their medications at the pharmacy on a monthly basis throughout the 1 ½ year duration of the study. The pharmacist will not only be responsible for educating subjects newly initiated on HAART but will be tracking a subject's ability to remain adherent to antiretroviral medications through counting unused pills monthly. Because a subject's ongoing relationship with the study will develop through the pharmacy, the pharmacist will ensure that study time-points such as specimen collection and knowledge assessment every 6 months are also met.

Coptic Hope pharmacy

The study pharmacy will dispense drugs to patients on behalf of the Coptic Hope pharmacy. The reason to dispense drugs through the study pharmacy is to ensure proper follow-up of study subjects and to conduct pill-counts that are not usually performed in the Hope pharmacy. The pharmacist should work closely with Dr. Mouheb at the Hope pharmacy to transfer antiretroviral medications and other drugs to the locked cabinet in the study pharmacy. An accurate inventory of drugs transferred between the Hope pharmacy and the study pharmacy must be maintained by the pharmacist. As per Dr. Mouheb, drug stocks at the study pharmacy can be replenished on a monthly basis or on the basis of need. All drugs acquired through the Hope pharmacy to be dispensed at the study pharmacy will be free of cost since they are either donated by the Government of Kenya or PEPFAR.

Pharmacy database

Inventory of drugs as well as monthly pill counts and patient drug history are to be recorded on the computer MS Access pharmacy database developed by Management Health Sciences (MHS) and customized by the data office. Every time a subject comes to

pick up their medications, the pharmacist should login into this database and fill it out accordingly. This is the same database that the Coptic Hope pharmacy uses with few modifications. These databases will be merged at the Hope data offices for record-keeping therefore every effort should be made to make the databases compatible in terms of drug accounting. The study pharmacy database contains an extra page of reporting pill counts that should be filled out at each monthly patient visit. This page of information is not included in the Hope pharmacy database. Any changes to the database should be coordinated with Dr. Mouheb at the Hope pharmacy and Charles in the data office.

HAART Initiation

When a patient is to initiate HAART for the first time, the pharmacist should check the Tracking form see that all steps have been conducted and checked prior to administration of drugs. If any of the steps have not been taken (for example, Arm A subjects have not undergone adherence counseling), then the pharmacist should not administer the drug but re-book the subject to complete the necessary steps. Please review chapter 4 to learn what steps subjects in various arms must undergo before initiation of HAART.

Most, if not all, patients will start a nevirapine-containing regimen. This means that patients will start nevirapine at half the regular dose and increase their dose 2 weeks after drug initiation. Therefore, patients should be requested to return after 2 weeks to increase the regimen to full dose.

At the first visit and each visit thereafter, the pharmacist should write down the next appointment for the subject in the “Pharmacy Followup tracking form” and fill in the “Followup” questionnaire. This information should be kept in the patient’s file and regularly scanned into a computer database by the Data clerk.

Follow-Up

Subjects should follow-up with the pharmacists on a monthly basis to pick up their refills of antiretroviral medications. Patients are required to return monthly and not longer. Under no circumstances are patients to be given prescription refills greater than one month in duration. Monthly visits to the pharmacy are necessary in order to perform pills counts which are the primary outcome of measurement in this study and therefore a requirement for participation.

The visits are also an important time to track the completion of Knowledge Assessment forms and Specimen Collection and Adherence #3. Knowledge assessment forms are administered by the laboratory assistant. The first assessment is done at enrollment. The next knowledge assessment is performed one month after initiating HAART. Thereafter, it is administered every 6 months from HAART initiation. It is the responsibility of the pharmacist to ensure that knowledge assessments are performed at the specified dates due, by tracking the subjects on the Pharmacy Follow-up Tracking form. Antiretroviral drugs are not to be administered until these knowledge assessments are performed.

At the same time as knowledge assessments at months 6, 12, and 18 after HAART initiation, blood specimens are to be collected from the subject. One vial of blood will be collected by the laboratory assistant immediately after or before the knowledge assessment form is filled out. Again, antiretroviral medications are not to be dispensed until specimens are collected on the specified dates after HAART initiation.

Those subjects randomized to receive Adherence counseling are in Arms A and C. The pharmacist should track these subjects closely to ensure that Adherence counseling #3 is conducted 6 weeks after initiating HAART. Therefore, the first month after starting the full-dose nevirapine regimen, the pharmacist should check that Adherence #3 has been performed before dispensing any medications.

Pre-Screening Talking Points for Clinical Staff

Dr. Michael Chung is conducting a research study at the Hope Center. This study examines how to help patients adhere to their medications by comparing different interventions. These patients who enroll in the study are followed over 18 months by Dr. Chung and other doctors from the University of Washington in America and the University of Nairobi. You appear to be eligible to be in this study and can possibly enroll in this trial if you like.

Participation in the research study is entirely voluntary and does not affect medical care at Hope in any way. Patients who decide not to participate continue to be treated at Hope. Patients who do participate are treated like any other patient except for the aspect of adherence counseling and promotion. This aspect of care is determined by the research study. Enrolled study patients also pick up their antiretroviral prescriptions from the Study Pharmacist and not the Hope Center pharmacist.

The study may help you by helping you take your medications better. But it may also not help you at all. Are you interested in learning more about the study from the Study Coordinator? She can explain the study in more detail if you are interested. If you are, we will send you to her right now. If not, I will continue to book you to see the other Hope Center staff.

Talking Points for Hope Center Staff

Dr. Michael Chung will be conducting a research study at the Hope Center. This study will examine how to help patients adhere to their medications by comparing different interventions. 400 adult patients will be enrolled in the study. They will be randomly assigned to 1 of 4 groups. Each group will contain 100 people. These patients will be followed over 18 months and will receive their medical care at the Hope Center.

Participation in the research study is entirely voluntary and will not affect medical care at Hope in any way. Patients who decide not to participate will continue to be treated at Hope. Patients who do participate will be treated like any other patient except for the aspect of adherence counseling and promotion. This aspect of care will be determined by the research study for the 1 ½ years these patients are enrolled in this study. Enrolled study patients will also pick up their antiretroviral prescriptions from the Study Pharmacist and not the Hope Center pharmacist.

Patients will be invited to enroll in the study if they are > 18 years old, are eligible to start antiretroviral medications, will allow home visits, and plan to live in Nairobi for at least 2 years.

Clinical officers who identify HAART treatment naïve adult patients who are eligible for treatment by CD4 count or WHO clinical staging will refer these patients to the Study Nurse before sending them to the HAART Protocol.

The Study Nurse will explain the study to the patient and invite them to join. If an individual does not want to participate the Study Nurse will continue them on the HAART protocol and they will remain at the Hope Center to receive antiretroviral treatment. If patients decide to join, then the Study Nurse will modify the adherence

counseling that they will receive. Subjects will be assigned to one of the following groups:

1. a group that will receive 3 education counseling sessions from a counselor about how to take anti-HIV medications correctly;
2. a group that will carry an electronic alarm device for six months to help remember when to take anti-HIV medications;
3. a group that will receive both education counseling and carry the alarm device for six months;
4. and a group that will receive neither intervention

All subjects will receive counseling from the Study Pharmacist. They will continue to receive medical care and free antiretroviral treatment at the Hope Center, the same as any other patient at the Hope Center. They will also see the nutritionist and the social worker. However, they will not be part of the standard Hope adherence counseling sessions.

The Study Nurse will be available at morning meeting everyday to discuss any questions you might have. She will also speak to clinical officers to discuss potential patients who might be referred to the study.

If you have any questions or concerns the Study Nurse can't answer, please contact Dr. Michael Chung at 675-1136.

Talking Points for Recruitment

You are being invited to participate in a research study at the Hope Center. The study is being conducted by Dr. Michael Chung and other doctors from the University of Washington in America and the University of Nairobi. The Hope Center is where the study will take place although the Coptic Hospital is not directly involved in the study. This study examines how to help patients take their anti-HIV medications correctly.

You do not have to join the study. Whether or not you join the study will not impact your care at the Hope Center in any way. If you are eligible, you will still receive free antiretroviral treatment and care from the Hope Center. If you do participate, you will continue to receive free antiretroviral treatment and care at Hope now and after the study is done. The main difference between the joining the study and not joining is the type of counseling and support you will receive to know when and how to take your medications correctly. 400 adult patients will be enrolled in the study and will be assigned to 1 of 4 groups. Each group will contain 100 people. If you decide to join the study, you will be put in one of these groups and followed for 1 ½ years in the study.

The study will also see where your home is located from clinic and the neighborhood where you live. We will do this to see if these things influence whether you take your medications correctly, to provide social support, and to trace you if you become lost to the study. We will also draw blood from you 4 times over 18 months in order to see how much HIV is in your blood and whether the adherence interventions are working for you.

You are referred to us because the clinical officer has determined that you need to start anti-HIV medications to treat your HIV disease.

- Do you plan to start anti-HIV medications as your doctor suggests?
- Are you over 18 years of age?
- Have you ever taken anti-HIV medications before?

If you are interested in the study, I will explain more about it from the informed consent form which I will give or read to you. If you still want to be in the study after reading or being read the informed consent, you can sign the form and we can enroll you in the study.

Counseling Education and Interventions

GUIDELINES FOR ADHERENCE COUNSELING (ARMS A AND C)

INTRODUCTION

At the conclusion of the first session of Adherence Counseling, patients will understand:

1. What HIV and AIDS are
2. What CD4 count is
3. What Antiretroviral Medications (ARVs) are
4. What the problem of Resistance is
5. What the problems of Side Effects are
6. What the importance of Adherence is
7. Whether they should start ARVs therapy

COUNSELOR DUTIES

- After you have explained the topics to the client, ask them to explain main points from each of the seven topics, in order to get a sense for their understanding of the information. Do this at the end of each section
- If you think they have understood the material well and are ready, schedule them for another appointment 1 week later for Adherence Counseling #2.
- Let the client reflect on the subject matter and give them time to decide whether ARVs are right for them.
- Schedule Adherence Counseling #3 one month after starting antiretroviral medications

Record ALL questions or comments the patient may have on Adherence Counseling questionnaires.

---

1. HIV/AIDS ☐

What Is HIV?

- HIV stands for “Human Immunodeficiency Virus”
- HIV is a virus that attacks the body’s immune system that protects the body from infections

How Is HIV Transmitted?

- Sexual contact
- Needles
- Exchange of blood and bodily fluids
- Mother-to-child transmission

What is the difference between HIV and AIDS?

- When a person’s CD4 count falls below 200 or they become very sick with opportunistic infections, then they are considered to have AIDS and not just HIV
- AIDS is an advanced stage of HIV infection that happens after a few years
- AIDS stands for “Acquired Immune Deficiency Syndrome”
- When a person has AIDS taking Antiretroviral Medications can help lessen the severity of the disease
- **What questions do you have about HIV/AIDS?**

2. CD4 COUNT ☐

What Are CD4 Cells?

- CD4 cells are immune cells that protect the body from infections
- CD4 cells prevent infections and keeps the body healthy

How do you measure CD4 Cells?

- The CD4 cells are counted by a blood test
- It is called a CD4 COUNT
- A normal CD4 count in an adult is above 500

What Happens to CD4 Cells When HIV Enters the Body?

- HIV attacks and destroys CD4 cells
- After years of constant attack from HIV, the CD4 count starts to fall

What happens when CD4 count falls?

- When the CD4 count falls below 200, diseases called ‘opportunistic infections’ are able to infect the body because the body can’t defend itself
- Opportunistic infections include: TB, pneumonia, skin problems, white spots in the mouth, and chronic diarrhea
- **What questions do you have so far?**

3. ANTIRETROVIRAL MEDICATIONS ☐

What are Antiretroviral Medications or ARVs?

- ARVs are a combination of 3 different kinds of strong medicine
- ARVs lower the amount of HIV virus in the blood
- When the virus level is low then the CD4 count can increase
- Increased CD4 count means the body is able to protect itself against opportunistic infections

What are the benefits of ARVs?

- After a few weeks of taking ARVs you begin to regain appetite and weight
- Many people report an increase in their energy levels and general sense of well being
- People can often then return to work or school or care for their families

When do you start ARVs?

- You begin ARV treatment when the immune system is so damaged by HIV that it no longer protects the body
- This is when you have AIDS (CD4 count less than 200 or severe opportunistic infections)

- Starting ARVs is never an emergency but should only be done after you receive education about the medications, understand how to take them, and are ready to begin.

Are ARVs a cure for HIV?

- No, ARVs are not a cure
- ARVs only lower the amount of virus so the body can protect itself from disease
- It cannot remove HIV completely from the body
- You still remain HIV-positive, but you can live a life free from AIDS and opportunistic infections

Can you still give HIV to others while you are taking ARVs?

- Yes, since HIV cannot be totally eliminated from the body, you can still transmit the virus others.
- You should therefore continue to be committed to prevention—practice safe sex (e.g., wear a condom whenever you have penetrative sex, practice methods of sex that are non penetrative, etc.)

How long do you take ARVs?

- ARV treatment is life-long
- You must take it everyday according to the schedule for the rest of your life
- You must take the dose at the scheduled time and never miss a dose, otherwise the treatment might fail and the virus becomes resistant
- **What questions do you have about ARVs or your regimen?**

4. RESISTANCE AND TREATMENT FAILURE ☐

What happens if you stop taking ARVs?

- When you stop taking ARVs the virus begins to produce to high levels again
- The level of virus goes back to the same high level it was before the medications were started

What happens if I don't take the medications regularly or take it off and on?

- The same thing: the virus begins to produce to high levels again

What happens if the virus returns to high levels?

- At high levels, the virus can change and become resistant to the ARVs
- When HIV becomes resistant, then the ARVs you are using no longer have an effect on the virus
- Resistance happens by not taking the medications correctly and by starting and stopping medication repeatedly
- When resistance occurs, this is called treatment failure

What happens in treatment failure?

- In treatment failure, the ARVs no longer work because the virus has become resistant.
- If treatment fails, then it is necessary to use stronger, more expensive medicines that may not work
- You may have worse side effects and not be able to afford these expensive medicines
- If you become resistant to these new ARVs then eventually you will run out of options and all ARVs will no longer work
- Thus it is ESSENTIAL that you are adherent to the ARVs and that you take them as you are supposed to in order to improve and maintain your health
- **Please explain back to me why it is important to take all of your medicines as much as possible.**

5. SIDE EFFECTS ☐

Are there side effects from taking ARVs?

- Yes, sometimes people can get side effects from taking ARVs
- Side effects vary from person to person

- Some people have none while others experience mild effects which are unpleasant but often manageable
- Most side effects occur within the first few weeks then improve after a few weeks or months
- Side effects can include:
    - Skin rash
    - Headache
    - Loss of appetite
    - Fatigue
    - Nausea, vomiting, and diarrhea
    - Muscle pain or numbness/tingling in the limbs or extremities
- Usually the side effects go away after some time, so if the side effects are mild it is important to continue taking the medications as prescribed and not to miss any doses. You can often treat mild side effects yourself with other medicines or diet. Ask your pharmacist or nutritionist about this.
- However, sometimes side effects can become so severe that you have to stop taking the ARVs and switch to a new regimen.
- It is important to let the doctor know immediately if you have severe side effects which means rash all over the body, constant vomiting or pain, and inability to eat or retain food. If the side effects are this severe, stop all ARVs and come to clinic right away.
- **What questions do you have about side effects?**

6. ADHERENCE ☐

How should you take ARVs?

- You must take the correct dosage. If you take less than the dose prescribed the treatment will not be effective and will result in resistance and treatment failure. Therefore, do NOT share ARVs with anyone.
- You must take ARVs at the right time of day. Most ARVs are taken twice a day. This means that you would take your medicines every 12 hours, *e.g.* 7:00AM and 7:00PM, according to the schedule of your daily activities
- If you are late taking your dose, you should follow the 6-hour rule: take your dose as soon as you remember up until 6 hours later. For example, if you take ARVs at 8:00AM and 8:00PM and you miss your 8:00AM dose, then you can take your dose up until 2:00PM (6 hours later). If it is after 2:00PM, then wait until the evening dose at 8:00PM to take the next dose. Remember that this is not the best solution and should be avoided.
- You must take ARVs according to any dietary restrictions. Some ARV medicines need to be taken with food; others need to be taken on an empty stomach. Ask the nutritionist what dietary restrictions there might be.
- It is essential to take ARVs exactly as prescribed and not miss doses.
- ARVs will only work when the amount of drug circulating in the body is kept at a certain level. For this to happen, you must take ARVs exactly as prescribed.
- Some other medications interact with ARVs and make them ineffective. Be sure to tell your doctor and pharmacist the names of all the medications (including traditional/herbal medicines) that you are taking as well as any new medications.

How can you make sure you take the ARVs as prescribed?

- Ask a friend, partner, or family member to remind you to take the medications
- Set a scheduled time in the day to take the ARVs
- Put up notes in the home or at work to remind you to take the medications

- Associate your doses with an event that occurs at the same time everyday. For example, when you eat breakfast and dinner.
- You can set dose reminders with an alarm clock, an alarm device, a watch, or cell phone.
- **Please tell me some ways you plan to help yourself remember to take ARVs.**

7. DECISIONS ABOUT STARTING ARVs ☐

What do you need to think about before starting ARVs?

- Deciding whether to start ARV treatment is your decision
- You should wait to begin therapy until you feel ready
- Take enough time to think the decision through and get as much information about ARVs as you need
- Feel free to ask many questions about ARVs to the doctor, pharmacist, or counselor
- Starting ARVs is never an emergency but should be done only after one has made a commitment to taking lifelong therapy

Ask yourself certain questions to determine if you are ready to start ARV therapy:

- Can I come every month to the HIV clinic/doctor's office for follow-up and medicine refills?
- Will I commit myself to tolerating the side effects for the sake of a better quality of life in the future?
- Do I have adequate support (*e.g.,* a family member or friend) who can help me adhere to the treatment?
- Can I meet the financial obligations for adherence to ARVs?
- Can I take the medications everyday according to the prescribed schedule?

GUIDELINES FOR PHARMACIST
ARMS A, B,C, and D: CONTROL

Basic caution on the possible adverse effects of antiretrovirals and the importance of maintaining therapy will be given by the pharmacist at the time of drug administration.

1. ADHERENCE ☐

How should you take ARVs?

- You must take the correct dosage. If you take less than the dose prescribed the drugs may not work. Therefore, do NOT share ARVs with anyone.
- You must take ARVs at the right time of day. Most ARVs are taken twice a day. This means that you would take your medicines every 12 hours, *e.g.* 7:00AM and 7:00PM, according to the schedule of your daily activities
- If you are late taking your dose, you should follow the 6-hour rule: take your dose as soon as you remember up until 6 hours later. For example, if you take ARVs at 8:00AM and 8:00PM and you miss your 8:00AM dose, then you can take your dose up until 2:00PM (6 hours later). If it is after 2:00PM, then wait until the evening dose at 8:00PM to take the next dose. Remember that this is not the best solution and should be avoided.
- It is essential to take ARVs exactly as prescribed and not miss doses.
- Some other medications interact with ARVs and make them ineffective. Be sure to tell your doctor and pharmacist the names of all the medications (including traditional/herbal medicines) that you are taking as well as any new medications.

Can you still give HIV to others while you are taking ARVs?

- Yes, since HIV cannot be totally eliminated from the body, you can still transmit the virus others.

- You should therefore continue to be committed to prevention—practice safe sex (e.g., wear a condom whenever you have penetrative sex, practice methods of sex that are non penetrative, etc.)

2. SIDE EFFECTS ☐

Are there side effects from taking ARVs?

- Yes, sometimes people can get side effects from taking ARVs
- Side effects vary from person to person. Some people have none while others experience mild effects which are unpleasant but often manageable
- Most side effects occur within the first few weeks then improve after a few weeks or months
- Side effects can include:
  - Skin rash
  - Headache
  - Loss of appetite
  - Fatigue
  - Nausea, vomiting, and diarrhea
  - Muscle pain or numbness/tingling in the limbs or extremities
- Usually the side effects go away after some time, so if the side effects are mild it is important to continue taking the medications as prescribed and not to miss any doses. You can often treat mild side effects yourself with other medicines or diet. Ask your pharmacist or nutritionist about this.

However, sometimes side effects can become so severe that you have to stop taking the ARVs and switch to a new regimen.

ENROLLMENT

Patient ID Number: __ __ __ **Interviewer number __ __ __ __**

Date of interview (day/month/year) ___ ___/___ ___/___ ___ ___ ___

1. SOCIODEMOGRAPHIC

Date of Birth (day/month/year) ___ ___/___ ___/___ ___ ___ ___
Age ____________ years
Is the subject male or female? (tick one) Male ☐ Female ☐
(1) How many years of school did you complete? ____________ years
(2) Highest education level completed? None ☐
Primary ☐
Secondary ☐
College ☐
What languages do you speak? (check all that apply)
Kikuyu ☐ Luhwa ☐ Kamba ☐
Luo ☐ Kalenjin ☐ Kisii ☐
Other ☐ (specify) ________________________________
Current marital status: Married (monogamous) ☐
Married (polygamous) ☐
Steady boyfriend/girlfriend ☐
Single ☐
Divorced/Separated ☐
Widowed ☐
Employment: Salaried job ☐
Self-employed ☐
Casual laborer ☐
Housewife ☐
Unemployed ☐
Do you have a steady partner? *yes* ☐ / *no* ☐
If yes, answer questions **H** through **L**. If no, skip to question **M.**
Do you live with your current partner? *yes* ☐ / *no* ☐
How long have you been with your current partner? (tick one)
☐ < 1 year ☐ 1 – 5 years ☐ 6 – 10 years ☐ > 10 years
Partner's employment: Salaried job ☐
Self-employed ☐
Casual laborer ☐
Housewife ☐
Unemployed ☐
Don't know ☐
Does your partner support you financially? *yes* ☐ / *no* ☐
What is the highest education level completed by your partner? None ☐
Primary ☐
Secondary ☐
College ☐
Don't know ☐
How many rooms are there in your house? (excluding toilets) _____

What type of toilet do you have? *(check one)* Pit-latrine ☐
Flush ☐
Do you share a toilet with people outside your household? *yes* ☐ / *no* ☐
How many people live in your house? ____________
What is your monthly rent? ____________ Ksh

3. TRAVEL AND TRANSPORTATION
How long does it take for you to travel to clinic?
How much does it cost to travel to and from clinic?
Do you walk from home to clinic? *yes* ☐ / *no* ☐
If no, what form of public transportation do you use and how describe in detail how you get to clinic from home (eg. bus route numbers)

2. MEDICAL HISTORY
Have you ever had any of these diseases?
Syphilis ☐
Gonorrhea ☐
Genital ulcers ☐
Shingles ☐
TB ☐
Oral lesions ☐
None ☐

3. SEXUAL HISTORY
At what age did you first have sexual relations? ____________ years
How many sexual partners have you had in your life? ____ number male
____ number female
When was the last time you received money or other favors in exchange for sex OR gave money or other favors in exchange for sex?
☐ within last month ☐ 1 – 12 months ago ☐ > 12 months ago ☐ never

DATA ENTRY (*tick*): ☐ DATE:____/____/____ SIGN: ____________

It is important to let the doctor know immediately if you have severe side effects which means rash all over the body, constant vomiting or pain, and inability to eat or retain food. If the side effects are this severe, stop all ARVs and come to clinic right away

ADDRESS AND INTAKE

Patient ID Number: __ __ __ **Interviewer Number:** ____ ____

Date of interview (day/month/year) ___ ___/___ ___/___ ___ ___ ___

Name (three names): ____________________ ________________________ ___________________

Current Residence: ____

Estate: ________________________ Plot Number: ________ Door Number:_____________

Directions to residence from Coptic Hope Center:
Route Number 1: _______ Route Number 2: _______ Route Number 3: _____
Disembark at: __________
Closest Landmarks (e.g. school, church, bar, butchery, etc.): ____

GPS address (longitude and latitude): ____

Place of work: ____

Postal address of work: ____

Physical address of work: ____

Spouse's name: ____

Spouse's place of work: ____

Spouse's occupation: ____

Postal address of spouse: ____

Physical address of spouse: ____

Primary Contact:

Phone Number (Cell): ___________________
Phone Number (Landline): ______________

Relationship to phone owner (tick one)

Self □ Husband or wife □ Relative □ Friend □ Employer □ Other (specify) □

Upcountry Contacts

Upcountry telephone contact

Contact person: ____
Relationship to patient (tick one):
Husband or wife □ Relative □ Friend □ Employer □
Other (specify) □

Phone Number (Cell): ___________________ Phone Number (Landline): ______________

Town: ____________________________________
Upcountry Postal Code (P.O. Box): _________________________________

GPS address (longitude and latitude): ____ ____
Upcountry physical address: ____ ____ ____

Secondary Contact (Significant Contact in Nairobi)

Contact person: ____

Relationship to patient (tick one):
Husband or wife □ Relative □ Friend □ Employer □
Other (specify) □

Phone Number (Cell): ___________________
Phone Number (Landline): ______________

Secondary Contact Postal Code (P.O Box):
Secondary Physical Address:

FOLLOW-UP

Patient ID Number: __ __ __ **Interviewer number** __ __

Date of interview (day/month/year) ___ ___/___ ___/___ ___ ___ ___

Is the patient taking antiretroviral medications? yes □ / no □
What is/are the antiretroviral medication(s)? (tick one)

d4T (30mg)-3TC-NVP
d4T (40mg)-3TC-NVP
d4T (30mg)-3TC-EFV
d4T (40mg)-3TC-EFV
d4T (30mg)-ddI-LPV/rit
AZT-3TC-EFV
AZT 3TC-NVP
AZT-ddI-LPV/rit
AZT-3TC-LPV/rit
d4T (40mg)-ddI-LPV/rit
Other Please Specify: _____________________

During the last 7 days, how many antiretroviral pills did the patient MISS taking?

□□

During the last 30 days, how many antiretroviral pills did the patient MISS taking? (

□□

If patient missed any doses, please specify reasons (check all that apply).

- □ Toxicity/side effect
- □ Share with others
- □ Forgot
- □ Felt better
- □ Too ill
- □ Stigma, disclosure or privacy issues
- □ Drug out of stock
- □ Patient lost or ran out of pills
- □ Delivery/travel problems
- □ Inability to pay
- □ Alcohol
- □ Depression
- □ Other (specify)

Did patient take the morning dose today? yes □ / no □
Did the patient take the evening dose today? yes □ / no □
Did the patient bring the pill bottle? yes □ / no □
If yes, how many pills found in bottle? □□

Do you have pills stored elsewhere (e.g., pill box) or are all the pills in the bottle? yes □ / no □
Have you shared your pills with anyone else? yes □ / no □
How many pills were given at this visit? □□

If the patient was given an alarm device, has the patient been using it?
yes □ / no □

If no, why?

When is the next appointment for followup? (dd/mm/yyyy) ________________

HOME ASSESSMENT

Patient ID Number: ___ ___ ___ ___ **Interviewer Number:** ____ ____

Today's Date (day/month/year) ___ ___ / ___ ___ / ___ ___ ___ ___

1) **Housing Conditions**
   a) Which estate does the client live in? ____
   b) How many rooms are in the house? ☐☐
   c) How many people live in the household? ☐☐
   d) What material is the floor made of? *(tick one)*
   ☐ Cement ☐ Earth ☐ Tiles ☐ Wood ☐ Other (specify)
   e) What material is the wall made of? *(tick one)*
   ☐ Stone ☐ Corrugated iron sheeting ☐Brick/block ☐ Grass/reeds ☐ Mud/wood
   ☐ Tin ☐ Mud/cement ☐ Wood only ☐ Other (specify)
   f) What material is the roof made of? *(tick one)*
   ☐ Corrugated iron sheeting ☐ Tiles ☐ Concrete ☐ Asbestos sheets ☐ Grass ☐ Makuti ☐ Tin ☐ Other (specify)
2) **Water Supply**
   a) What is the general water availability in the estate? *(tick one)*
   Good ☐ Fair ☐ Bad ☐
   b) What is the water source for the family? *(tick one)*
   Piped into the house ☐ Communal tap within the housing units ☐
   Water tank in compound ☐ Communal water tank within vicinity ☐
   No water source within the vicinity ☐ Any other source (specify __________) ☐ Water from a river, a pool or any other open water body source ☐
3) **Sanitation** *(tick one)*
   Own flush toilet in the house ☐ Communal flush toilet in the housing units ☐
   Pit latrine for the family only ☐ Communal pit latrines in the housing units ☐
   No toilet or pit latrine in vicinity ☐
4) **Source of Energy for Cooking** *(tick one)*
   Gas ☐ Charcoal ☐ Electricity ☐
   Wood ☐ Paraffin ☐ Other (specify ______) ☐
5) **GPS**
   Waypoint number ___________ Accuracy ________ meters
   GPS Location S01. ____________________________________
   E036. ____________________________________
   Were you standing at the entrance of the home when measuring? *yes* ☐ / *no* ☐
   If no, how many meters were you standing away from entrance? ______ meters
   Location Name: ____
   Physical Address: ____
   Directions to residence: ____
   ____
   ____

____
___
Useful local landmarks (e.g. name of nearby school, church, bar, butchery, etc.): _____________
____
____
Comments: ____

HOME TRACING

ID Number: ___ ___ ___ **Interviewer Number: ____ ____**

Date of visit (day/month/year) ___ ___ / ___ ___ / ___ ___ ___ ___

1. House found? *yes ☐ / no ☐*
2. Subject found? *yes ☐ / no ☐*

   If not, specify person found ____
3. Subject sick? *yes ☐ / no ☐*
4. Specify why subject stopped coming to clinic: *(check whichever is applicable and give details)*

   Subject illness ☐

   Family illness ☐

   Upcountry visit ☐

   Transport problem ☐

   Employment related ☐

   Partner objected ☐

   Other family member(s) objected ☐

   Other (specify) ☐_____________________________
5. Is subject attending another clinic *yes ☐ / no ☐ / don't know ☐*

   If yes, specify __________________________________
6. Does subject plan to return to the Hope Center? *yes ☐ / no ☐ / don't know ☐*
7. Appointment date given *yes ☐ / no ☐*

   If yes, indicate date __ __/__ __/__ __ __ __
8. Comments:

____

____

____

____

KNOWLEDGE ASSESSMENT

Date of Assessment (day/month/year): _________________ Patient ID number: _____

1. I know when all of my HIV medications are supposed to be taken.
   - ☐ Strongly Agree
   - ☐ Somewhat Agree
   - ☐ Somewhat Disagree
   - ☐ Strongly Disagree

2. I know what to do if I miss a dose of any of my HIV medications (For example, whether or not to take it later).
   - ☐ Strongly Agree
   - ☐ Somewhat Agree
   - ☐ Somewhat Disagree
   - ☐ Strongly Disagree

3. Taking HIV medications correctly can reduce a person's viral load.
   - ☐ Strongly Agree
   - ☐ Somewhat Agree
   - ☐ Somewhat Disagree
   - ☐ Strongly Disagree

4. If I don't take my HIV medications as directed, these kinds of medications may not work for me in the future.
   - ☐ Strongly Agree
   - ☐ Somewhat Agree
   - ☐ Somewhat Disagree
   - ☐ Strongly Disagree

5. I know what the possible side effects of my HIV medications are.
   - ☐ Strongly Agree
   - ☐ Somewhat Agree
   - ☐ Somewhat Disagree
   - ☐ Strongly Disagree

6. I know how to handle side effects that I could get (or have gotten) from my HIV medications.
   - ☐ Strongly Agree
   - ☐ Somewhat Agree
   - ☐ Somewhat Disagree
   - ☐ Strongly Disagree

7. Skipping a few HIV medication pills every week would not really damage my health.
   - ☐ Strongly Agree
   - ☐ Somewhat Agree
   - ☐ Somewhat Disagree
   - ☐ Strongly Disagree

8. Taking a break from my HIV medication every so often should help and not hurt my health.
   - ☐ Strongly Agree
   - ☐ Somewhat Agree
   - ☐ Somewhat Disagree
   - ☐ Strongly Disagree

9. If I am feeling healthy, it is pretty much a sure sign that I am taking my HIV medication consistently enough.
   - ☐ Strongly Agree
   - ☐ Somewhat Agree
   - ☐ Somewhat Disagree
   - ☐ Strongly Disagree

10. If I am feeling good, I don't really need to take my HIV medications.
    - ☐ Strongly Agree
    - ☐ Somewhat Agree
    - ☐ Somewhat Disagree
    - ☐ Strongly Disagree

11. Taking combinations of HIV medications on schedule helps keep the right amount of medication in a person's system.
    - ☐ Strongly Agree
    - ☐ Somewhat Agree
    - ☐ Somewhat Disagree
    - ☐ Strongly Disagree

12. Certain combinations of HIV medications must be taken with or without meals in order to be fully effective.
    - ☐ Strongly Agree
    - ☐ Somewhat Agree
    - ☐ Somewhat Disagree
    - ☐ Strongly Disagree

13. "Resistance" to a particular HIV medication means it no longer works to kill the virus.
    - ☐ Strongly Agree
    - ☐ Somewhat Agree
    - ☐ Somewhat Disagree
    - ☐ Strongly Disagree

LOSS TO FOLLOW-UP

Patient ID Number: ___ ___ ___ **Interviewer Number:** ____ ____

Date (day/month/year) ___ ___ / ___ ___ / ___ ___ ___ ___

1. Date of last clinic visit (day/month/year) ___ ___ / ___ ___ / ___ ___ ___ ___
2. Date last seen by staff (day/month/year) ___ ___ / ___ ___ / ___ ___ ___ ___
3. Source of information *(check all that apply))*

   Subject ☐

   Partner ☐

   Family member ☐

   Friend ☐

   Study clinician/staff ☐

   Neighbor ☐

4. Reason for loss to follow-up (*check all that apply)*:

   No longer willing to participate ☐

   (Provide details) ________________________________

   Moved ☐

   Suspected death ☐

   Confirmed death ☐

   Other ☐

   (Provide details) ________________________________

5. Unable to trace *yes ☐ / no ☐*
6. Comments:

____

____

____

DATA ENTRY *(tick):* ☐ DATE: ____/____/____ SIGN: ________________

MORTALITY

Patient ID Number: __ __ __ **Interviewer number __ __ __ __**

Date of interview (day/month/year) ___ ___/___ ___/___ ___ ___ ___

Date of death (day/month/year) ___ ___ / ___ ___ / ___ ___ ___ ___

Age at death _____

Last CD4 count ___________ Date specimen obtained __ __/__ __/__ __ __ __
dd mm yy

Cause of death *(tick all that apply)*:

- Pneumocystis pneumonia ☐
- Toxoplasmosis ☐
- Meningitis, Cryptococcal ☐
- Meningitis, Other ☐
- Kaposi's Sarcoma ☐
- Non-Hodgkin's Lymphoma ☐
- AIDS dementia ☐
- Sepsis ☐
- Meningitis ☐
- Encephalopathy ☐
- Pneumonia (bacterial) ☐
- Tuberculosis ☐
- Malaria ☐
- Typhoid fever ☐
- Accidental ☐
- Unknown ☐
- Other (specify) ☐

____

The informant source for the cause of death was: *(tick one)*

- Partner/spouse ☐
- Parent ☐
- Friend ☐
- Hospital records/staff ☐

Other (specify) ☐ ________________________________

8. The cause of death is: *(tick one)*

HIV related ☐

Non-HIV related ☐

Indeterminate ☐

Unknown ☐

Note:

Probable **HIV related deaths** are if:

a) A person who meets the above criteria for AIDS

OR

b) A person dies from a severe infection

Probable **non-HIV related deaths** are if:

a) A person does not meet criteria for AIDS **AND** does not die from a severe infection

OR

b) A person dies an accidental death

Indeterminate deaths are all deaths not included in the above two categories

Unknown deaths are those for which no information is available

DATA ENTRY *(tick)* ☐ DATE: ____/____/____ SIGN: ___________

ADHERENCE COUNSELING #1

ID Number: ___ ___ ___ ___ **Interviewer Number:** ____ ____

Today's Date (day/month/year) ___ ___ / ___ ___ / ___ ___ ___ ___

Checklist	Tick
Explain about HIV and how it infects the body	
Explain about CD4 cells and why it is necessary to measure the CD4 count	
Explain the difference between HIV and AIDS	
Explain about ARV	
Explain that ARV is not a cure	
Explain cause of resistance	
Explain treatment failure	
Explain importance of adherence	
Explain problem of side effects	
Have patient think about life long commitment of therapy	
Have patient think about ability to followup care	
Explore patient support system	
Discuss adherence promotion strategies	

Identify barriers to adherence (tick all that apply)

- ☐ Poor communication
- ☐ Inadequate understanding about HIV/AIDS
- ☐ Failure to disclose status
- ☐ Mental state
- ☐ Stigma
- ☐ Lack of social support
- ☐ Alcohol/drug use
- ☐ None
- ☐ Other (specify)
- ☐ Low literacy

Does the patient need to move forward with the protocol or repeated Adherence Counseling #1?

- ☐ Move forward
- ☐ Repeat Adherence Counseling #1

Notes/remarks:

ADHERENCE COUNSELING #2

ID Number: ___ ___ ___ ___ **Interviewer Number:** ____ ____

Today's Date (day/month/year) ___ ___ / ___ ___ / ___ ___ ___ ___

Score Scale: 1-Very poor 2-Poor 3-Fair 4-Good 5-Excellent

Question	Rationale	Score
What do you know about ARV?	Assess whether information given in Counseling Adherence #1 has been understood.	
Do you know the names of any ARV?	Assess whether client knows that AZT, Nevirapine, etc. are ARV, but Septrin is not.	
Do you know how ARV works?	Assess client's knowledge of basic ARV action (especially that ARV is not a cure for HIV infection).	
Do you know what side effects of ARV are there and what to do if you have side effects?	Assess client's knowledge of side effects related to his/her ARV regimen and the appropriate response to deal with side effects	
Do you know how long you should normally take ARV?	Assess whether client knows that ARV is life-long treatment.	
Do you know what happens if you don't take your ARV consistently?	Assess whether client understand the problem of resistance given ARV interruptions	
Do you know the purpose of CD4 counts?	Assess whether client knows that CD4 count is a laboratory indicator for monitoring the effect of ARV.	
Are you still interested in taking ARV?	Assess whether client continues to be motivated to begin treatment.	
If yes, what are your expectations from ARV?	Assess whether client has realistic expectations, e.g., prolonging life, keeping them well enough from their family, etc. Assess for false expectations, e.g., a cure for HIV, etc.	
Assess for factors that help determine capability for follow up	Assess whether client can attend HIV clinic for follow up medical and counseling care.	
Ask the client whether s/he feels ready for long term treatment; if s/he can come to HIV clinic for regular follow up.	Assess commitment for long-term treatment.	
Ask the client whether s/he has a relative/friend whom he can rely on to support him take ARV.	Assess availability of support at home.	
Ask patient if s/he has barriers to adherence	Assess barriers to adherence and progress made	

On a scale of 1 to 10 (10 being most ready, 1 being least), please rate the client's ability to adhere to medications.

Least likely **Likely** **Most likely**

1 2 3 4 5 6 7 8 9 10

Does the patient need to move forward with the protocol or to repeat Adherence Counseling #1?

☐ Move forward ☐ Repeat Adherence Counseling #1

Notes/remarks:

ADHERENCE COUNSELING #3

To be repeated one month after starting ARVs

ID Number: ___ ___ ___ ___ **Interviewer Number:** ____ ____

Today's Date (day/month/year) ___ ___ / ___ ___ / ___ ___ ___ ___

Score Scale: 1-Very poor 2-Poor 3-Fair 4-Good 5-Excellent

Question	Rationale	Score
What do you know about ARV?	Assess whether information given in Counseling Adherence #1 has been understood.	
Do you know the names of any ARV?	Assess whether client knows his/her medication and dosage	
Do you know how ARV works?	Assess client's knowledge of basic ARV action (especially that ARV is not a cure for HIV infection).	
Do you know what side effects of ARV are there and what to do if you have side effects?	Assess client's knowledge of side effects related to his/her ARV regimen and the appropriate response to deal with side effects	
Do you know how long you should normally take ARV?	Assess whether client knows that ARV is life-long treatment.	
Do you know what happens if you don't take your ARV consistently?	Assess whether client understand the problem of resistance given ARV interruptions	
Do you know the purpose of CD4 counts?	Assess whether client knows that CD4 count is a laboratory indicator for monitoring the effect of ARV.	
How are you taking your ARVs?	Assess whether client continues to be motivated to begin treatment.	
If yes, what are your expectations from ARV?	Assess whether client has realistic expectations, e.g., prolonging life, keeping them well enough from their family, etc. Assess for false expectations, e.g., a cure for HIV, etc.	
Assess for factors that help determine capability for follow up	Assess whether client can attend HIV clinic for follow up medical and counseling care.	
Ask the client whether s/he feels ready for long term treatment; if s/he can come to HIV clinic for regular follow up.	Assess commitment for long-term treatment. What will help?	
Ask the client whether s/he has a relative/friend whom he can rely on to support him take ARV.	Assess availability of support at home. If none exists, did you think of other options?	
Ask client whether s/he sees the need for continued prevention.	Assess review need for continued prevention e.g. condom use	

During the last 7 days how many ARV pills did the patient MISS taking? (tick one)

☐ 0 ☐ 1 ☐ 2 ☐ 3 ☐ 4 ☐ 5 ☐ 6 ☐ 7 ☐ ≥8

On a scale of 1 to 10 (10 being most ready, 1 being least), please rate the client's ability to adhere to medications.

Least likely **Likely** **Most likely**

1 2 3 4 5 6 7 8 9 10

Does the patient need to move forward with the protocol or to repeat Adherence Counseling #1?

☐ Move forward ☐ Repeat Adherence Counseling #1

Notes/remarks:

Questionnaire Definitions

General Information

In this section, basic instructions are provided on how to complete the questionnaires. After reading the following section and you want further clarification please direct your questions to the data manager and principal investigator.

Delivering the Questionnaire Questions

Read the questions **exactly** as they are written in the questionnaires. Closely follow the instructions of each section when asking the questions.

After reading a question in a clear and comprehensible manner, wait quietly for a response. If the respondent does not answer in the reasonable time, he has probably 1) not heard the question; or 2) not understood the question; or 3) does not know the answer. If there is no answer, repeat the question. If there is still no reply, you must ask whether the question has been understood. If the answer is 'No', you may reword (rephrase) the question. If the difficulty lies in finding the right answer, you should help the respondent to consider his/her reply. Interact with the client as much as possible.

Recording responses

The response received from the respondent should be written on the questionnaire in *blue or black biro pen*. Responses written in pencil can easily become smudged and difficult for the scanner to read and interpret. Also do NOT use other funny pens/ biro pens or fountain pens other than the above stated pens.

Responses **should** be written clearly in upper-case letters.

Correcting mistakes

If a mistake is made in the recording of a response, do not erase the response using *white out.* Please circle the wrong answer and strike through the wrong answer once. After this initial and date the mistake on the form. Then check (tick) the right answer or write the correct response next to the question.

Example:

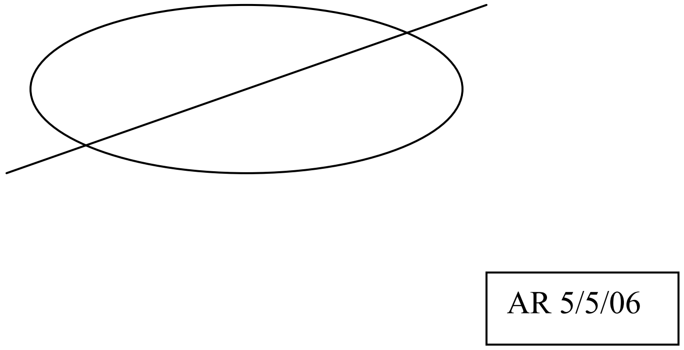

Write clearly

Always write very legibly in capital (BLOCK) letters. This instruction is particularly important for the Address and Intake, because the names and locations of the client are the most important connecting points with the patient. If the response is not legible, the data clerk nor the scanner will be able to work with the information.

NOTE: SHOULD YOU EXPERIENCE PROBLEMS WHILE FILLING THE FORM PLEASE CONSULT WITH THE DATA MANAGER.

Enrollment Form

Study ID number

This is the unique research identification number that is given by the study coordinator at the time the subject enters the research study.

Interviewer number

This is a unique code given to all interviewers in the study staff. If you do not have an interviewer numbers please contact the data manager.

Date of the interview

Provide the current date that the interview is taking place. Information on dates should be recorded as day (dd), month (mm), year (yyyy).

Q1: Date of birth:

Ask for the date of birth when the client was born. For older individuals who may have trouble determining the month and day of birth complete as much of the information on the birth date as you are able to. If unknown, please complete the information as accurately as possible. For example if the subject knows the year, fill in the year that he or she was born. If the date of birth is not stated or unknown then code “01” for day, “01” for month and “9999” for year. Also include in a note on the side that the patient did not know the day, month or year of birth.

Q2: Age (year):

Ask how old the patient is in years. If this does not correspond to the date of birth, please re-state the question to the subject. Record the answer as the subject states it in the box, make a note on the side of the questionnaire that the response does not agree with date of birth.

Q3. Gender:

Ask the sex of the individual. Do not use the name of the individual to assume the sex of that individual. However, some caution in asking this is important not to offend the respondent.

Q4: How many years of school did you complete?

The question should be interpreted as years of school and not include years of school repeated.

Q5: Highest education level completed?

Tick one of the following answers (none, primary, secondary, college) according to the subjects highest education level completed.

Q6: What languages do you speak (check all that apply)?

Check all the languages that the subject speaks locally. If the subject can speak French, English, or German check others and place language in the box.

Q7: Current Martial Status

As of the day of enrollment what is the martial status of the subject.

Married (monogamous) – is the subject married formally

Married (polygamous) – is the subject married to multiple partners formally

Steady boyfriend/girlfriend – is the subject have a regular partner

Single – the subject is not currently in a relationship

Divorced/Separated – the subject was previously in a marriage

Widowed – the subject was in a marriage until their partner passed away

Q8: Employment

Ask the subject what current occupation.

Salaried – does the subject get regular payment for their work

Self-employed – does the subject own has his/her own business, sets his/her own hours, makes their own products, ect.

Casual Laborer – is the subject currently in a short-term employment; ie house keeper

Unemployed – currently the subject is not working

Q9: Do you have a steady partner?

Please tick yes or no if the subject is currently has a steady partner.

Q10: How long have you been with your current partner?

Tick the box that corresponds to subjects length of time with current partner

Q11: Partner's Employment:

Ask the subject what current occupation of his or her partner.

Salaried – does the subject get regular payment for their work

Self-employed – does the subject own has his/her own business, sets his/her own hours, makes their own products, ect.

Casual Laborer – is the subject currently in a short-term employment; ie house keeper

Unemployed – currently the subject is not working

Don't know

Q12: Does your partner support you financially?

Checking Yes, signifies that the partner provides support of food, shelter, or other material goods.

Q13: What is the highest education level completed by your partner?

Tick one of the following answers (none, primary, secondary, college) according to the subject's partner highest education level completed. Check 'don't know' if subject doesn't know

Q14: How many rooms are there in your house? (excluding toilets)

Place the number of rooms there are in the subjects house, if the patient does not know or refuse to answer place '99' in the boxes. If the patient is homeless put '00' as the number of rooms in there house.

Q15: What type of toilet do you have? (check one)

Check if the subject as a 'pit-latrine' or a 'flush' toilet that they use on a regular basis – whether they live in a home or not.

Q16: Do you share a toilet with people outside your household?

Check yes if the subject states that people outside the immediate household use the same toilet as subject.

Q17: How many people live in your house?

Put the number of people that live/or share the subjects place of living currently. This should include the subject.

Q18: What is your monthly rent? (Ksh)

Place how much the subject spends on his/her housing per month. If the subject does not know put '999999' or if the subject owns their home put '000000'. In addition circle the answer and add the answer on the side of the question.

Q19: How long did it take to travel to patient's house? Hh/mm

Ask the patient how long it took the patient to get clinic 'that day' -- place the number of hours and minutes in the designated boxes.

Q20: How much did it cost for 1 person to get to patients house (ksh)

Place the amount of money was spent by the subject to get to clinic on that day.

Q21: Do you walk from home to clinic?

On that day, did the subject walk to clinic from his/her home – Yes or No.

Q22: Have you ever had any of these diseases? (check all that apply)

Ask if the subject has had any of the following disease individually and check all that apply. If the subject has any questions about the disease the study coordinator should answer all questions or concerns. Check none if the subject has never had any of the disease listed in the question.

Q23: At what age did you first have a sexual relationship? (years)

Place the age at which the subject states as having his/her first sexual relationship.

Q24: How many sexual partner have you had in your life?

Place the number of male sexual partners in the male section, and the number of female partners in the female section. Do not leave the boxes blank, if the subject has had zero female partners but '00'.

Q25: When was the last time you received or given money or other favors in exchange for sex;

Read the answer to subject and tick the one that corresponds to patients answer.

IF THE PATIENT REFUSES TO ANSWER ANY QUESTION, CIRCLE QUESTION AND STATE THE PATIENT REFUSED TO ANSWER THE QUESTION

Address and Intake Form

Study ID number

This is the unique research identification number that is given by the study coordinator at the time the subject enters the research study.

Hope ID number

This is the unique identification that is given to the subject at the Hope Center for Infectious disease

Interviewer number

This is a unique code given to all interviewers in the study staff. If you do not have an interviewer numbers please contact the data manager.

Date of the interview

Provide the current date that the interview is taking place. Information on dates should be recorded as day (dd), month (mm), year (yyyy).

Name

List the names of the client starting with the sir name first, and then followed by the other names of the patient. List all the names of the client without leaving out any. For example if a patient is called Makambong'o John Paul Otieno and his sir name is Otieno then record as follows

O	T	I	E	N	O		J	O	H	N		P	A	U	L		M	A	K	A	M	B	O	N	G	‘	O		

The name you are known as in your neighborhood

This is the name that the subject is known among the local community

Current Residence

The home address of the subject

Estate

Local region/neighborhood the subject lives in

Plot number

Record if the subject as number to identify his/her place of residence

Door number

Record the subject door number on his or her residence.

Directions to residence from Coptic

Route Number

Refers to the number on the busses or matatu used to get to the subjects home from Coptic Hospital.

Disembark at

At what station/area/street does the subject get dropped from

Closest Landmark

Include the specific names of schools, churches, bars, and butcheries that close to the subjects place of residency

Place of Work

Name of the place/company where the subject works

Primary Contact

If the subject was lost or ill, what is the number we should try to contact the subject

Upcountry Contact

If the subject spends anytime with contact outside of Nairobi please include this information in this area

Secondary Contact

If the subject was lost of sick, a second contact person that could be used to find the subject

Home Assessment Form

This questionnaire is primarily filled by the community health workers

Study ID number

This is the unique research identification number that is given by the study coordinator at the time the subject enters the research study.

Interviewer number

This is a unique code given to all interviewers in the study staff. If you do not have an interviewer numbers please contact the data manager.

Date of the interview

Provide the current date that the interview is taking place. Information on dates should be recorded as day (dd), month (mm), year (yyyy).

Clients Home

Q1: How long did it take to travel to patient's house?

The CHW should record how much time in hours (hh) and minutes (min) to get to the subjects house from leaving the research clinic.

Q2: How much did it cost for 1 person to get to patients house from clinic

The CHW should record the cost it took to get to the subjects house.

Q3: How many matatu transfers were needed – record number and include the numbers in the route numbers

Housing Conditions

Q4: Which estate does the client live in?

Q5: How many rooms are in the house?

-Do not include closet space but include kitchen or washrooms.

Q6 and Q7: What material is the wall and roof made of?

Water Supply

Q8: What is the general water availability? (tick one)

Good – greater 90% of time water is available

Fair – around 75% of the time water is available

Bad -- less than 50% of the time water is available

Q9: What is the water source for the family?

Sanitation

Source of Energy for cooking

GPS -- These should be recorded by the Garman GPS devices

Waypoint

Accuracy (meters)

GPS Location (S01)

(E036)

Be as specific as possible when it comes to describing the location, physical address, directions to residence and useful local landmarks.

Follow-up Form

This questionnaire is primarily filled by the pharmacy technologist

Study ID number

This is the unique research identification number that is given by the study coordinator at the time the subject enters the research study.

Interviewer number

This is a unique code given to all interviewers in the study staff. If you do not have an interviewer numbers please contact the data manager.

Date of the interview

Provide the current date that the interview is taking place. Information on dates should be recorded as day (dd), month (mm), year (yyyy).

Q: Is the patient taking antiretroviral medications?

-Currently (Today) is the patient taking antiretroviral medications – yes or no

Q: What are the antiretroviral medications the subject is taking?

Q: During the last 7 days how many antiretroviral pills did the patient MISS taking?

-Record the NUMBER of pills that the subject remembers missing

-This is self-report data

Q: During the last 30 days, how many antiretroviral pills did the patient MISS taking?

-Record the NUMBER of pills that the subject remembers missing

-This is self-report data

Q: If the patient missed any doses, please specify reason (check all that apply)?

-Drug out of stock

-Depression – refers to the patient feeling sad

Q: Did the patient bring the pill bottle?

-Please remind the subject that the he/she much bring the bottle every time they come to clinic

Q: How many pills were given at this visit?

-Include the total number of pills that were given at the current visit

Q: When is the next appointment for follow-up?

-This must be given an every follow-up visit.

Home Tracing Form

This questionnaire is primarily filled by the community health worker

Study ID number

This is the unique research identification number that is given by the study coordinator at the time the subject enters the research study.

Interviewer number

This is a unique code given to all interviewers in the study staff. If you do not have an interviewer numbers please contact the data manager.

Date of the interview

Provide the current date that the interview is taking place. Information on dates should be recorded as day (dd), month (mm), year (yyyy).

Q: Specify why subject stopped coming to clinic (tick all that apply)?

-Subject Illness (Was the subject ill or hospitalized – please include details in the box)

-Family Illness (Include who and with what illness if possible in box)

-Upcountry Visit

-Transport problem

-Employment Related

-Partner objected

Q: Was an appointment date given?

-If the subject is found and would like to return to research – call the office to setup an appointment

Knowledge Assessment Form

This questionnaire is primarily filled by the lab technologist.

The purpose of this form is to assess the patient's subjective knowledge about antiretroviral medications and HIV.

Study ID number

This is the unique research identification number that is given by the study coordinator at the time the subject enters the research study.

Interviewer number

This is a unique code given to all interviewers in the study staff. If you do not have an interviewer numbers please contact the data manager.

Date of the interview

Provide the current date that the interview is taking place. Information on dates should be recorded as day (dd), month (mm), year (yyyy).

Loss To Follow-up Form

This questionnaire is primarily filled by the Study Coordinator

The purpose of this form is to provide information on subject that have not returned to clinic.

Study ID number

This is the unique research identification number that is given by the study coordinator at the time the subject enters the research study.

Interviewer number

This is a unique code given to all interviewers in the study staff. If you do not have an interviewer numbers please contact the data manager.

Date of the interview

Provide the current date that the interview is taking place. Information on dates should be recorded as day (dd), month (mm), year (yyyy).

Date of last clinic visit

This information should be obtained from subjects research file

Date last seen by staff

This information should be obtained from study personnel

Mortality Form

This questionnaire is primarily filled by the study coordinator.

Study ID number

This is the unique research identification number that is given by the study coordinator at the time the subject enters the research study.

Interviewer number

This is a unique code given to all interviewers in the study staff. If you do not have an interviewer numbers please contact the data manager.

Date of the interview

Provide the current date that the interview is taking place. Information on dates should be recorded as day (dd), month (mm), year (yyyy).

Date of Death

This should be the date of death according to family, community, or hospital

Age at Death

Last Cd4 count

This information should be kept on Laboratory Forms from Hope Center File

Cause of Death

This information should be obtained from medical records

STUDY CALENDAR TEMPLATE

Research ID	Hope ID	Visit Purpose	Date of Actual Attendance	Interviewer signature

<u>Visit Purposes</u>

Adherence 1

Adherence 2

Adherence 3

Clinician Scheduled

Clinician PRN

Lab Results

General Counseling

Social work

Nutritionist

Specimen Collection

Other (explain)

STUDY PHARMACY CALENDAR

Knowledge Assessment

Pharmacy Followup

Specimen Collection

Pharmacy Follow-up

Date scheduled	Form	Date confirmed	Interviewer signature	Data Entry Signature	Comments and Queries
___/___/___	PHARMACY FOLLOWUP (2 week step-up)	___/___/___			
___/___/___	PHARMACY FOLLOWUP *** (Month 1)	___/___/___			
___/___/___	KNOWLEDGE ASSESSMENT *** (Month 1)	___/___/___			
___/___/___	PHARMACY FOLLOWUP (Month 2)	___/___/___			
___/___/___	PHARMACY FOLLOWUP (Month 3)	___/___/___			
___/___/___	PHARMACY FOLLOWUP (Month 4)	___/___/___			
___/___/___	PHARMACY FOLLOWUP (Month 5)	___/___/___			
___/___/___	PHARMACY FOLLOWUP (Month 6) ***	___/___/___			
___/___/___	KNOWLEDGE ASSESSMENT *** (Month 6)	___/___/___			
___/___/___	SPECIMEN *** COLLECTION #2 (Month 6)	___/___/___			
___/___/___	PHARMACY FOLLOWUP (Month 7)	___/___/___			
___/___/___	PHARMACY FOLLOWUP (Month 8)	___/___/___			
___/___/___	PHARMACY FOLLOWUP (Month 9)	___/___/___			
___/___/___	PHARMACY FOLLOWUP (Month 10)	___/___/___			

**Date scheduled**	**Form**	**Date confirmed**	**Interviewer signature**	**Data Entry Signature**	**Comments and Queries**
___/___/___	PHARMACY FOLLOWUP (Month 11)	___/___/___			
___/___/___	PHARMACY FOLLOWUP *** (Month 12)	___/___/___			
___/___/___	KNOWLEDGE ASSESSMENT *** (Month 12)	___/___/___			
___/___/___	SPECIMEN *** COLLECTION #3 (Month 12)	___/___/___			
___/___/___	PHARMACY FOLLOWUP (Month 13)	___/___/___			
___/___/___	PHARMACY FOLLOWUP (Month 14)	___/___/___			
___/___/___	PHARMACY FOLLOWUP (Month 15)	___/___/___			
___/___/___	PHARMACY FOLLOWUP (Month 16)	___/___/___			
___/___/___	PHARMACY FOLLOWUP (Month 17)	___/___/___			
___/___/___	PHARMACY FOLLOWUP *** (Month 18)	___/___/___			
___/___/___	KNOWLEDGE ASSESSMENT *** (Month 18)	___/___/___			
___/___/___	SPECIMEN *** COLLECTION #4 (Month 18)	___/___/___			

ARM A (Adherence Counseling)

Form	Date Attended	Interviewer signature	Data Entry Signature	Comments and Queries
INFORMED CONSENT	____/____/____		N/A	
ADDRESS & INTAKE	____/____/____			
ENROLLMENT	____/____/____			
KNOWLEDGE ASSESSMENT	____/____/____			
SPECIMEN COLLECTION #1	____/____/____			
HOME ASSESSMENT	____/____/____			*Date Scheduled:*
ADHERENCE #1	____/____/____			*Date Scheduled:*
ADHERENCE #2	____/____/____			*Date Scheduled:*
PHARMACY COUNSELING	____/____/____		N/A	
HAART INITIATION	____/____/____		N/A	
ADHERENCE #3	____/____/____			*Date Scheduled:*

ARM B (Alarm Device)

Form	Date Attended	Interviewer signature	Data Entry Signature	Comments and Queries
INFORMED CONSENT	____/____/____		N/A	
ADDRESS & INTAKE	____/____/____			
ENROLLMENT	____/____/____			
KNOWLEDGE ASSESSMENT	____/____/____			
SPECIMEN COLLECTION #1	____/____/____			
HOME ASSESSMENT	____/____/____			*Date scheduled:*
ALARM HANDOUT	____/____/____		N/A	
PHARMACY COUNSELING	____/____/____		N/A	
HAART INITIATION	____/____/____		N/A	
STOP ALARM DEVICE	____/____/____		N/A	*Date Scheduled:*

ARM C (Adherence Counseling & Alarm Device)

Form	Date Attended	Interviewer signature	Data Entry Signature	Comments and Queries
INFORMED CONSENT	____/____/____		N/A	
ADDRESS & INTAKE	____/____/____			
ENROLLMENT	____/____/____			
KNOWLEDGE ASSESSMENT	____/____/____			
SAMPLE COLLECTION #1	____/____/____			
HOME ASSESSMENT	____/____/____			*Date Scheduled:*
ADHERENCE #1	____/____/____			*Date Scheduled:*
ADHERENCE #2	____/____/____			*Date Scheduled:*
ALARM HANDOUT	____/____/____		N/A	
PHARMACY COUNSELING	____/____/____		N/A	
HAART INITIATION	____/____/____		N/A	
ADHERENCE #3	____/____/____			*Date Scheduled:*
STOP ALARM DEVICE	____/____/____		N/A	*Date Scheduled:*

ARM D (Control)

Form	Date Attended	Interviewer signature	Data Entry Signature	Comments and Queries
INFORMED CONSENT	____/____/____		N/A	
ADDRESS & INTAKE	____/____/____			
ENROLLMENT	____/____/____			
KNOWLEDGE ASSESSMENT	____/____/____			
SPECIMEN COLLECTION #1	____/____/____			
HOME ASSESSMENT	____/____/____			*Date scheduled:*
PHARMACY COUNSELING	____/____/____		N/A	
HAART INITIATION	____/____/____		N/A	

University of Washington & University of Nairobi
STUDY WRITTEN CONSENT FORM

Antiretroviral Adherence Interventions: A Randomized Trial

Study Investigators:
Michael H. Chung, MD, MPH, Acting Instructor, Department of Medicine, University of Washington, 675-1136
Grace John-Stewart, MD, PhD, Associate Professor, Department of Medicine, University of Washington, 011-206-543-4278
James Kiarie, MBChB, MMed, MPH, Honorary Lecturer, Department of Obstetrics and Gynecology, University of Nairobi, 271-4159
Jane Simoni, PhD, Associate Professor, UW Department of Psychology, 011-206-685-3291
Jonathan Mayer, PhD, Professor, Department of Geography, University of Washington, 011-206-543-7110
Julie Overbaugh, PhD, Member, Fred Hutchinson Cancer Research Center, 011-206-667-3524
Barbra Richardson, PhD, Research Associate Professor, Department of Biostatistics, University of Washington, 011-206-731-2425

Emergency telephone number staffed 24 hours a day: 272-0831 or 272-4737
Ethical Review Committee Chairperson: Professor K. M. Bhatt, 272-6300

Researchers' Statement

You are being asked to join this research study of HIV/AIDS because you are > 18 years old, are coming to the Hope Center for care, are eligible to start taking anti-HIV medications for the first time, and will allow home visits. You are also being asked to allow storage of samples of your blood that are not used immediately for this study but will be used for future studies of HIV/AIDS. The people in charge of this study are named above.

The purpose of this consent form is to give you the information you will need to help you decide whether to be in the study or not. Please read the form carefully. You may ask questions about the purpose of the research, what we would ask you to do, the possible risks and benefits, your rights as a volunteer, and anything else about the research or this form that is not clear. When we have answered all your questions, you can decide if you want to be in the study or not. This process is called 'informed consent.' If you wish, we will give you a copy of this form for your records. Deciding whether or not to be in the study will not affect your ability to receive medical care and treatment from the Hope Center in any way.

Purpose of the Study

The goal of this study is to learn the best way to help people starting anti-HIV medications for the first time to take these medications correctly. This study will compare whether education counseling, an alarm device, both education counseling and the alarm, or neither of these is better to help people take their medications.

Procedures

<u>Group Assignment</u>

If you are eligible and agree to join this study, you will be randomly assigned to one of four groups:

- a group that will receive 3 education counseling sessions from a counselor about how to take anti-HIV medications correctly;
- a group that will carry an electronic alarm device for six months to help remember when to take anti-HIV medications;

- a group that will receive both education counseling and carry the alarm device for six months;
- and a group that will receive usual care but neither alarm device nor education counseling

Random assignment is like "flipping a coin", you have an equal chance of being in any one of these groups. We won't know which intervention you will receive until we open the envelope that has a sheet of paper telling us which intervention you will get. Neither you nor we choose your intervention. Rather, the envelopes contain papers assigning the interventions and we can't know until we open the envelope which one you will be assigned to. About 400 women and men will take part in this study, and 100 people will be in each group.

Interventions

- In the education counseling intervention, you will meet with a counselor who will counsel and educate you on HIV/AIDS and the importance of taking anti-HIV medications. You will meet with the counselor twice before you start taking anti-HIV medications and once after you start. The first two sessions will occur approximately one week apart and last approximately 45 minutes each. The third session will occur one month after starting medications and will last approximately 45 minutes. These sessions will occur here at the Hope Center. The counselor will make sure you know how to take the medications properly, and will explore with you different ways to improve your adherence to anti-HIV medications.
- In alarm device intervention, you will be given an alarm device that will be individually programmed to your medication schedule. It is a small device that will fit in your pocket. At the time you are required to take the medications, the alarm will beep until you shut it off. You will be asked to carry the pocket alarm device at all times and use it to remember when to take your medications. You will use the alarm for 6 months after you begin taking anti-HIV medications, then you will return the alarm to the study. You will continue to be followed in the study after you return the alarm to see if you can still remember when to take the medications without depending on the alarm.
- All groups will receive counseling on how to take anti-HIV medications from the pharmacist. The group which receives neither educational counseling nor alarm device will still receive information on use and side effects of the drugs from the Study Pharmacist. Those people in the groups which receive 1) only the alarm device or 2) neither alarm device nor education counseling; will not receive the additional medication counseling that they would receive if they were only seen at the Hope Center and not enrolled in the study.

First Visits

At the beginning of the study, you will be:

- asked to tell us where you live and how we may contact you in Nairobi and your upcountry home
- asked to complete a series of questions (a survey) that will include your health, sexually activity, and knowledge of how to take anti-HIV medications
- accompanied home by a counselor to collect information about your living conditions and distance from clinic. We will do this to see how these things may affect your ability to take anti-HIV medications, to give you social support at

home, and to be able to trace you if you do not return to the research clinic. Your home location will be put into an electronic mapping device (GPS).

- given one 10-minute counseling session with the pharmacist who will explain side effects associated with the medications and the importance of taking them properly

Follow-up Visits

Once you enter this study, we will ask you to take part for 18 months.

You will be asked to return every month to pick up your anti-HIV medications from the study pharmacist. You will be asked to bring your pill bottle with you and the pharmacist will count the number of pills you take by hand or electronically. Also, when you pick up your medications, someone from the study will ask about your adherence to your anti-HIV medications in a 5-minute interview. One month after starting anti-HIV medications and then every 3 months thereafter, we will also assess your knowledge of anti-HIV medications in a 10-minute interview.

Blood samples

At the first visit, 6 months, 12 months, and 18 months after you enter the study:

- you will have about 7 ml (about 1.5 teaspoons) of blood drawn by study staff that will be stored. This blood samples may be sent to Seattle, U.S.A. to test for the amount of HIV virus in your blood and antiretroviral resistance unless viral testing and analyses are available locally
- in total, 4 vials of blood will be removed over 18 months for study purposes
- some of the blood will be stored for future HIV-related studies and will not be used for immediate testing. All names and identifying information will be removed after completion of the study in January 2011.

Hope Center

You will continue to receive medical care and antiretroviral treatment at the Hope Center now and after the study is complete. Your HIV medical care will be handled by Hope Center physicians and not by this research study. They will give you medications and may order other laboratory tests as they see fit. We will have access to your medical records at the Hope Center and will follow your medical course on anti-HIV medications. We will dispense the anti-HIV medications that the Hope Center physicians prescribe. If you have any medical problems, you will contact the Hope Center for care and treatment.

Risks and discomforts of being in the study

The study may collect personal information from you that may be embarrassing to talk about and may cause some distress or discomfort. For example, you may be asked how many sexually partners you have had. You do not have to answer any questions you do not wish. If any distress occurs, you will see one of our nurse counselors. As part of the study, you may meet other patients from this clinic whom you know from outside the clinic. We will be collecting blood samples from you using a needle and syringe. The puncture of the needle may be uncomfortable and leave a bruise. We will also accompany you to visit your home to assess how you live and where your home is located. Having someone visit you in this way may make you

feel uncomfortable or draw undesired attention from neighbors. There is also a possibility of invasion of privacy from the alarm devices if you are in one of the groups that receives the alarm.

Alternatives to Participation

You may choose not to be in the study. If so, you will continue to receive anti-HIV medications and medical care from the Hope Center. You will also still receive counseling on anti-HIV medications from both the pharmacist and the adherence counselor at the Hope Center in at least three educational sessions. You may also choose to receive treatment and testing from other doctors, local clinics, and hospitals in Nairobi. The study staff will discuss all options available to you.

Benefits of the Study

You may benefit from this study by increasing your ability to take your anti-HIV medications correctly. You may also receive no direct benefit from this study. The results of this study may show ways to help people take anti-HIV medications correctly.

Other information

Information about your diagnosis is confidential and we will keep your records in a locked office. Information about your diagnosis and your participation in the research will be available to you and to the study team but not to anyone outside of the study. Data and blood samples obtained from you will be kept indefinitely, but all identifying information on data and blood samples will be removed after completion of the study in January 2011. Some blood will be kept for future studies in HIV/AIDS. In order to be in the study, you must agree to collection and storage of your blood for testing.

There is no cost to you for participating in the study. You do not have to pay for any study visits, testing, counseling, or the alarm device. You will also not receive any money for participating in this study.

You may refuse to participate or may withdraw from the study at any time without penalty or loss of benefit to which you are otherwise entitled. Your relationship with staff and services at Hope Center for Infectious Diseases will not be affected in any way if you do or do not participate or if you enter the program and withdraw later.

The following groups may need to review study records about you: Institutional oversight review offices at the research site, the University of Washington, or state and federal regulators.

Questions about the study or any adverse events should be addressed to this investigator, the study nurse, or Dr. Michael Chung. Do you have any questions? Do you agree to participate?

Signature of investigator________________________________ Date _______________

Printed name of investigator________________________________

Subject's statement

This study has been explained to me. I volunteer to take part in this research. I have had a chance to ask questions. If I have questions later about the research, I can ask one of the researchers listed above. If I have questions about my rights as a research subject, I can call the Ethical Review Committee at Kenyatta National Hospital 272-6300. I will receive a copy of this consent form if I would like one.

Signature of subject __________________________________ Date _________________

Printed name of subject_____________________________________

copies to: Investigator and Subject

Data Safety and Monitoring Plan

The length of study enrollment is projected to be 1 year, and it is planned that CD4 counts and HIV-1 viral analyses will be collected every 6 months for each patient who will be followed for 1 ½ years. Since viral investigations may be done in Seattle, it may take another 6 months before these tests are performed. Given the variation in time between enrollment, collection, and testing of specimens, statistical comparisons of CD4 counts and HIV-1 viral loads between the two groups will be analyzed at 3 objective time points throughout the 5 year life of the study. At first when 25% of the testing data is available for analysis, second when 50% of the data is available and third when 75% of the data is available. If analyses show statistically significant and consistent differences in CD4 counts and HIV-1 viral loads between any two arms in the study over a period of a year, then the primary and co-investigators of the study will meet to discuss stopping or revising the interventions. This data and safety monitoring plan has been formalized as part of the study protocols, and a committee has been formed.
